# Shielding the Next Generation: Symbiotic Bacteria from a Reproductive Organ Protect Bobtail Squid Eggs from Fungal Fouling

**DOI:** 10.1128/mBio.02376-19

**Published:** 2019-10-29

**Authors:** Allison H. Kerwin, Samantha M. Gromek, Andrea M. Suria, Robert M. Samples, Dister J. Deoss, Kerry O’Donnell, Salvatore Frasca, Deanna A. Sutton, Nathan P. Wiederhold, Marcy J. Balunas, Spencer V. Nyholm

**Affiliations:** a Department of Molecular and Cell Biology, University of Connecticut, Storrs, Connecticut, USA; b Division of Medicinal Chemistry, Department of Pharmaceutical Sciences, University of Connecticut, Storrs, Connecticut, USA; c Department of Chemistry, University of Connecticut, Storrs, Connecticut, USA; d Mycotoxin Prevention and Applied Microbiology Research Unit, National Center for Agricultural Utilization Research, Agricultural Research Service, U.S. Department of Agriculture, Peoria, Illinois, USA; e Department of Comparative, Diagnostic, and Population Medicine, College of Veterinary Medicine, University of Florida, Gainesville, Florida, USA; f Department of Pathology and Laboratory Medicine, Fungus Testing Laboratory, University of Texas Health Science Center, San Antonio, Texas, USA; Johns Hopkins Bloomberg School of Public Health

**Keywords:** host-microbe symbioses, *Euprymna scolopes*, chemical defense, interaction-driven molecular networking, defensive symbiosis

## Abstract

The importance of defensive symbioses, whereby microbes protect hosts through the production of specific compounds, is becoming increasingly evident. Although defining the partners in these associations has become easier, assigning function to these relationships often presents a significant challenge. Here, we describe a functional role for a bacterial consortium in a female reproductive organ in the Hawaiian bobtail squid, Euprymna scolopes. Bacteria from the accessory nidamental gland (ANG) are deposited into the egg jelly coat (JC), where they are hypothesized to play a defensive role during embryogenesis. Eggs treated with an antibiotic cocktail developed a microbial biomass primarily composed of the pathogenic fungus Fusarium keratoplasticum that infiltrated the JC, resulting in severely reduced hatch rates. Experimental manipulation of the eggs demonstrated that the JC was protective against this fungal fouling. A large proportion of the bacterial strains isolated from the ANG or JC inhibited F. keratoplasticum in culture (87.5%), while a similar proportion of extracts from these strains also exhibited antifungal activity against F. keratoplasticum and/or the human-pathogenic yeast Candida albicans (72.7%). Mass spectral network analyses of active extracts from bacterial isolates and egg clutches revealed compounds that may be involved in preventing microbial overgrowth. Several secondary metabolites were identified from ANG/JC bacteria and egg clutches, including the known antimicrobial lincomycin as well as a suite of glycerophosphocholines and mycinamicin-like compounds. These results shed light on a widely distributed but poorly understood symbiosis in cephalopods and offer a new source for exploring bacterial secondary metabolites with antimicrobial activity.

## Abstract (precis)

Organisms must have strategies to ensure successful reproduction. Some animals that deposit eggs protect their embryos from fouling/disease with the help of microorganisms. Although beneficial bacteria are hypothesized to contribute to egg defense in some organisms, the mechanisms of this protection remain largely unknown, with the exception of a few recently described systems. Using both experimental and analytical approaches, we demonstrate that symbiotic bacteria associated with a cephalopod reproductive gland and eggs inhibit fungi. Chemical analyses suggest that these bacteria produce antimicrobial compounds that may prevent overgrowth from fungi and other microorganisms. Given the distribution of these symbiotic glands among many cephalopods, similar defensive relationships may be more common in aquatic environments than previously realized. Such defensive symbioses may also be a rich source for the discovery of new antimicrobial compounds.

## INTRODUCTION

Defensive symbioses are found in a number of host-microbe associations, wherein secondary metabolites derived from beneficial symbionts are often used to inhibit other microorganisms or to protect the host from predation (1–6). This phenomenon has been well studied in insect associations, for example, in a number of beetle species, termites, fungus-farming ants, and in the protection of beewolf larvae (6, 7). Many organisms lay their eggs in an environment where successful embryogenesis depends on minimizing fouling by microorganisms. Aquatic organisms are especially susceptible to fouling because their eggs are under constant exposure to high densities of microorganisms, including over 10^6^ bacterial cells/ml and 10^3^ fungal spores/ml in most coastal seawater (8). Given that cephalopod embryogenesis can often take weeks to months (9–11) and that biofilms can generally form in a matter of hours to days, mechanisms must be present to prevent microbial growth on externally laid eggs.

**FIG 1:**
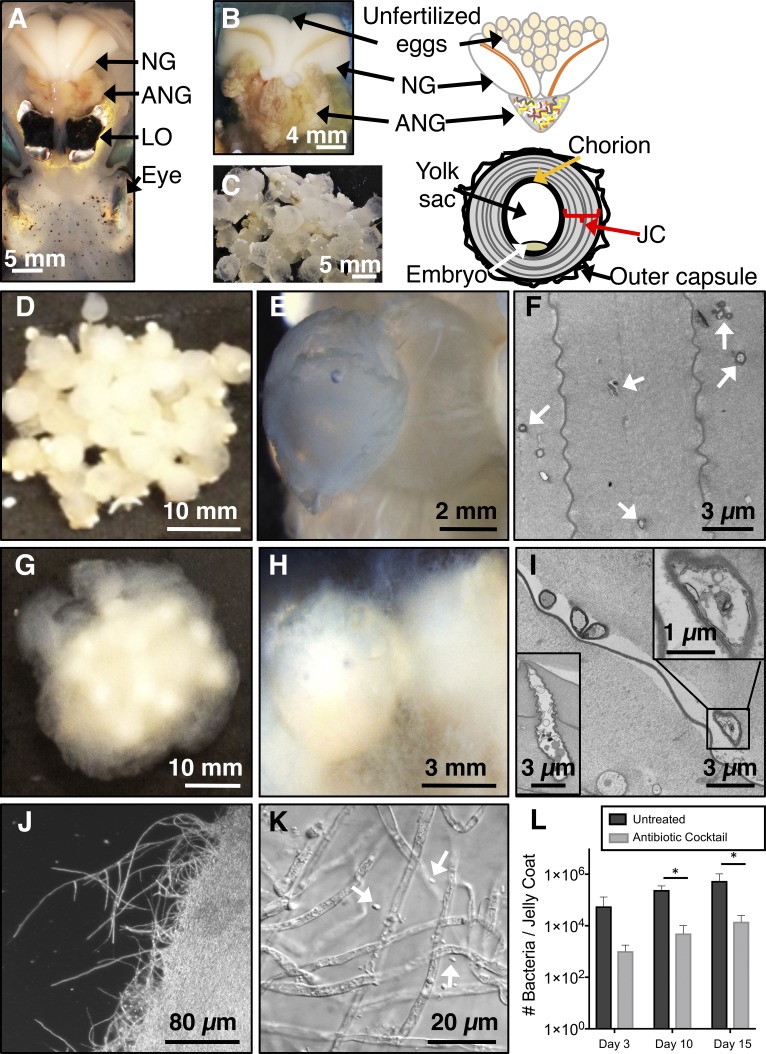
Antibiotic treatment of eggs leads to fungal fouling. (A) Ventral dissection of a female E. scolopes squid showing locations of reproductive system organs relative to the light organ and eye of the squid. (B and C) Detailed images and diagrams of female reproductive tract (B) and egg (C). Treatment of clutches with an antibiotic cocktail led to the formation of fungal/bacterial fouling. (D to I) Clutches were either left untreated (D to F) or were treated with antibiotics (G to I; *n* = 17). (J and K) The fungal biomass was composed of hyphae interspersed with bacterial cells (white arrows). (F) Electron micrographs of JCs from eggs at day 21 of embryogenesis show that untreated egg JCs contained both single bacterial cells and small clusters of cells (white arrows). (I) The antibiotic-treated egg JCs contained numerous fungal conidia and hyphae (insets). (L) After 3 days of treatment, a 98% reduction in bacterial load was observed (*t*_8_ = 1.685, *P = *0.131, *n* = 5 clutches). The bacterial load of both the untreated and the antibiotic-treated clutch segments increased over embryogenesis, but a significant reduction in bacteria was found both at day 10 (*t*_8_ = 5.011, *P = *0.001) and at day 15 (*t*_8_ = 2.511) (*P = *0.036; 98% and 97%, respectively; data in graph presented as means ± standard errors of the means [SEM]). NG, nidamental gland; ANG, accessory nidamental gland; JC, jelly coat; LO, light organ.

Many female cephalopods maintain a symbiotic bacterial community within their reproductive system in an organ known as the accessory nidamental gland (ANG; Fig. 1A and B) (12). The ANG community is composed predominantly of *Alphaproteobacteria*, *Gammaproteobacteria*, and *Verrucomicrobia*, depending on the cephalopod species (13–18). These bacteria are added to the egg jelly coat (JC; Fig. 1C) prior to clutch deposition on the substrate, after which the bacterial community composition is typically stable through embryogenesis (17). As the eggs do not receive parental protection, they face potential threats from fouling microorganisms and/or predation. Many hypotheses have been proposed to explain the functional role of the ANG bacteria, including assisting in sexual maturation of the host (19), contributing to formation of the egg capsule (12, 20), and providing protection for the developing embryos against pathogenic or fouling organisms (21). However, while the antibacterial activity of a few specific ANG/JC bacterial isolates and the whole ANG has been described previously (22, 23), the function of this association remains uncharacterized.

Demonstrating function in host-microbe associations can prove difficult, necessitating tractable experimental models and diverse approaches (24). The Hawaiian bobtail squid, Euprymna scolopes, offers just such an experimental model for studying symbiotic interactions. E. scolopes is well known for its association with the light organ symbiont Vibrio fischeri and, more recently, for its ANG consortium (17, 25). The host is easily maintained in the laboratory with minimal effect on the composition of the ANG bacterial community (16, 17), which is dominated by *Alphaproteobacteria* from the *Rhodobacteraceae*, as well as *Verrucomicrobia*, with some *Flavobacteriia* and *Gammaproteobacteria* (16–18). The ease of maintaining the host in the laboratory and the stability of its ANG community in captivity and throughout embryogenesis (17) make this system ideal for investigating the function of ANG bacteria in cephalopods.

Here, we present evidence for a role of the ANG bacterial consortium in host egg defense, integrating knowledge of community composition, functional ecology, analytical chemistry, and natural product discovery to begin to understand the role of bacterial members within the symbiosis. Using antibiotic treatment and egg manipulation, we demonstrate that ANG bacteria from E. scolopes provide resistance to fouling fungi. In addition, the majority of bacterial strains isolated from the ANG or eggs, as well as extracts from those strains, inhibited the fungus Fusarium keratoplasticum and/or the yeast Candida albicans. Interaction-driven molecular network analyses revealed metabolites with known antimicrobial activity, indicative of their possible role in the symbiosis.

## RESULTS AND DISCUSSION

### Antibiotic treatment of eggs leads to fungal fouling.

**FIG 2:**
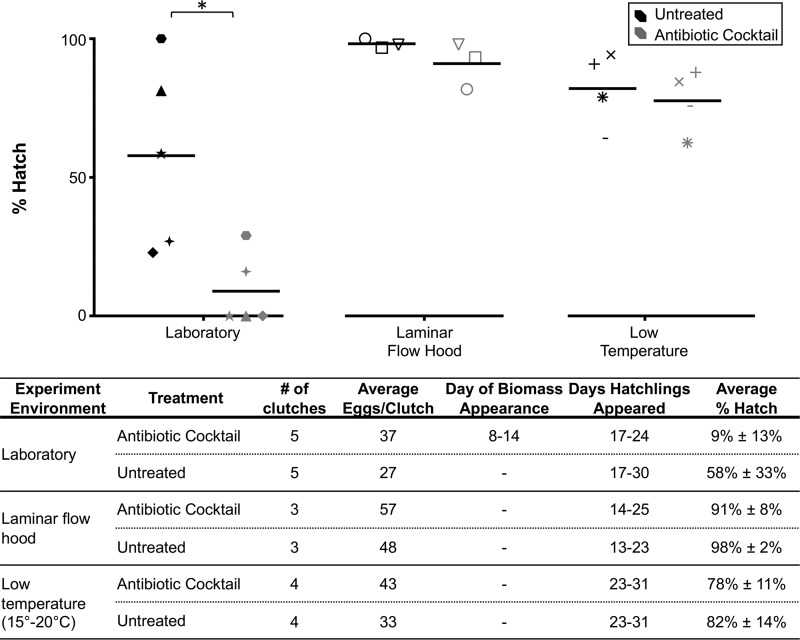
Treatment of eggs with an antibiotic cocktail and subsequent development of fungal fouling significantly reduced hatch of juveniles. Eggs treated with an antibiotic cocktail developed a fungal biomass between days 8 and 14 of embryogenesis and had a reduced hatch rate (*t*_4_ = 3.572, *P = *0.023). Hatching rates were unaffected when eggs were treated with the antibiotic cocktail and maintained in a laminar flow hood (*t*_2_ = 1.289, *P = *0.326) or at low temperatures (15 to 20°C, *t*_3_ = 0.738, *P = *0.514) to prevent fungal growth. The antibiotic cocktail included penicillin G, kanamycin, spectinomycin, streptomycin, and gentamicin, each at a concentration of 25 μg/ml. Matching data point shapes represent eggs taken from the same initial clutch.

To gain insights into the function of the JC bacteria, eggs were treated with an antibiotic cocktail (penicillin G, kanamycin, spectinomycin, streptomycin, and gentamicin; see Materials and Methods). The bacterial load in untreated eggs was similar to what had previously been described (17), while treated clutches experienced reduction in JC bacterial loads by 98% throughout embryogenesis (Fig. 1L) and developed heavy biofouling (Fig. 1G and H). Untreated clutches kept under the same conditions did not develop any biofouling (Fig. 1D and E). Egg biofouling resulted in very low hatch rates averaging 9% compared to 58% for untreated clutches (*t*_4_ = 3.572, *P = *0.023; Fig. 2). The fungal biomass was dominated by hyphae interspersed with bacterial cells (Fig. 1J and K). Fungal fouling visible by eye typically appeared on antibiotic-treated clutches between days 8 and 14 of embryogenesis (*n* = 17 clutches; Fig. 1G and H). However, fungal hyphae appeared on the antibiotic-treated egg surface as early as day 3 of embryogenesis (see Fig. S1B in the supplemental material) and developed into heavy biofouling by day 10 (Fig. S1H). The expanding fouling completely enveloped the clutch by day 15 (Fig. S1I to J). In contrast, no fungal hyphae appeared on untreated eggs from the same clutch at any point during development (Fig. S1E to G and K to M). Microconidia and hyphae were found dispersed throughout the JC layers but did not penetrate the chorion of antibiotic-treated eggs (Fig. 1I). The antibiotic-treated JCs also contained fewer bacterial cells than the JCs of untreated eggs (Fig. 1F, I, and L). When eggs were treated with the antibiotic cocktail at a lower temperature of 15 to 20°C (to inhibit fungal growth; see Materials and Methods) or under sterile conditions, hatching success was similar to that seen with untreated eggs (Fig. 2). The resulting juveniles were also successfully colonized with the light organ symbiont Vibrio fischeri. Thus, treatment with the antibiotic cocktail did not affect embryonic development or posthatching bacterial light organ colonization. Treatment with chloramphenicol alone (not present in the cocktail) led to the formation of a fungal biomass similar to that observed with treatment using the antibiotic cocktail but negatively influenced hatching success (Fig. S2).

10.1128/mBio.02376-19.1 FIG S1 A time series illustrating biofouling development over the course of embryogenesis. Clutches either were treated with antibiotic cocktail (B to D and H to J) or were left untreated (E to G and K to M). (A) Time series images focus on the surface of 1 or 2 eggs, such as the segment indicated by the red box. On day 3, a few hyphae were discernable (B, white arrow); those hyphae were found to have increased in number on days 6 (C) and 8 (D), with increased fouling during days 10 (H), 13 (I), and 15 (J). Untreated eggs showed no signs of fouling or fungal hyphae by day 15 (A and M). The same grain of sand can be seen in panels C and D, indicated by yellow arrows. Untreated eggs are also shown throughout embryogenesis, including day 3 (E), day 6 (F), day 8 (G), day 10 (K), day 13 (L), and day 15 (M). Representative images of untreated and antibiotic-treated eggs were taken from the same original clutch. Download FIG S1, PDF file, 0.2 MB. Copyright © 2019 Kerwin et al. 2019 Kerwin et al. This content is distributed under the terms of the Creative Commons Attribution 4.0 International license.

10.1128/mBio.02376-19.2 FIG S2 Chloramphenicol treatment of egg clutches resulted in fungal biomass. Eggs that were treated with 20 μg/ml of chloramphenicol dissolved in ethanol (*t*_6_ = 20.47, *P < *0.0001) or with 25 μg/ml of chloramphenicol dissolved in FSASW (*t*_4_ = 4.668, *P = *0.01) developed fungal fouling in both cases. Treatment with ethanol alone did not significantly reduce juvenile hatching (*t*_4_ = 2.33, *P = *0.08). However, hatching levels were affected when eggs were treated with chloramphenicol and maintained in a laminar flow hood (*t*_2_ = 103.6, *P <* 0.0001) to prevent fungal growth, indicating that chloramphenicol itself interfered with embryo development. Other studies have shown that juvenile squid and adults are not affected by chloramphenicol at similar doses (76–78). Data point shape reflects eggs taken from the same initial clutch. Download FIG S2, PDF file, 0.3 MB. Copyright © 2019 Kerwin et al. 2019 Kerwin et al. This content is distributed under the terms of the Creative Commons Attribution 4.0 International license.

Fungal isolates from the fouled clutches were analyzed via multilocus sequence typing (MLST) and were found to represent haplotype 2g of Fusarium keratoplasticum, a recently described species nested in the Fusarium solani species complex (FSSC; Fig. S3) (26). Fusarium keratoplasticum, together with at least 20 species in the FSSC, has been implicated in opportunistic infections of humans and other animals (27–30). Many of these persistent infections are difficult to treat because members of the FSSC and other fusaria are broadly resistant to currently available antifungal drugs (31).

10.1128/mBio.02376-19.3 FIG S3 Phylogeny of fungal isolates. One of three most parsimonious trees (MPTs) inferred from portions of three loci used to identify species and 3-locus haplotypes in the Fusarium solani species complex. Arabic numerals and lowercase roman letters, respectively, identify species and unique haplotypes. Numbers above internodes represent the percent frequency that they were recovered from 1,000 maximum likelihood (ML) and maximum parsimony (MP) bootstrap (BS) pseudoreplicates of the data. The ML-BS value is shown only where it differed by ≤5% from the MP-BS score. Bold internodes are used to identify eight genealogically exclusive in-group species, which were rooted on sequences of Fusarium lichenicola based on more-inclusive analyses (29). The three strains of F. keratoplasticum used in the present study are identified by gray highlight. CI, consistency index; PIC, parsimony informative characters; RI, retention index. Download FIG S3, PDF file, 0.6 MB. Copyright © 2019 Kerwin et al. 2019 Kerwin et al. This content is distributed under the terms of the Creative Commons Attribution 4.0 International license.

Eggs treated with a pulse of antibiotic cocktail and maintained in flowing Hawaiian seawater also developed fungal fouling dominated by F. keratoplasticum, demonstrating that this fungus was present and capable of fouling clutches under conditions similar to the host’s natural habitat. Four fungal isolates from experiments conducted using Hawaiian seawater were identified as F. keratoplasticum haplotype FSSC-2i (Fig. S3).

Profiling of the bacterial community of the fungal biomass that formed on the eggs during antibiotic treatment revealed that it was dominated by *Gammaproteobacteria* and *Alphaproteobacteria*, along with nine other bacterial classes (Fig. S4A). These fouling-associated bacteria were distinct from a previous analysis of the ANG/JC communities (Fig. S4B) (17), and the bacterial community compositions also differed widely between samples (Fig. S4C). Therefore, these fouling-associated bacteria likely consisted of environmental bacteria for which the fungal biomass provided a suitable substrate, but some of these might also have represented opportunistic pathogens of the eggs.

10.1128/mBio.02376-19.4 FIG S4 Bacterial diversity in fungal biomass. (A and B) The fungal biomass bacterial community (*n* = 12) was dominated by *Gammaproteobacteria* (A) and was distinct from that found in the ANG and JC (B). (C) The relative abundances of taxa that make up the fungal biomass community differed substantially between samples. Taxa are presented at the finest level obtained as follows: c, class; o, order; f, family; g, genus. Mean percentages of sequences/sample are represented by thick bars; standard deviations are represented by thin bars. Scatterplots are presented on a log scale to demonstrate variation for taxa present at lower average abundances. “Other” includes taxa present at <1% of the average fungal biomass as follows: unclassified *Bacteria*, *Stramenopiles* (o), *Pirellulaceae* (f), unclassified *Proteobacteria* (p), *Myxococcales* (o), and *Nannocystaceae* (f). An NMDS plot based on the Bray-Curtis metric of beta diversity demonstrated that the community composition of the fungal biomass is distinct from that of the ANG/JC (B). ANG and JC data were previously published (17). Download FIG S4, PDF file, 0.5 MB. Copyright © 2019 Kerwin et al. 2019 Kerwin et al. This content is distributed under the terms of the Creative Commons Attribution 4.0 International license.

### Symbiont-containing egg JC is protective against fungal fouling.

**FIG 3:**
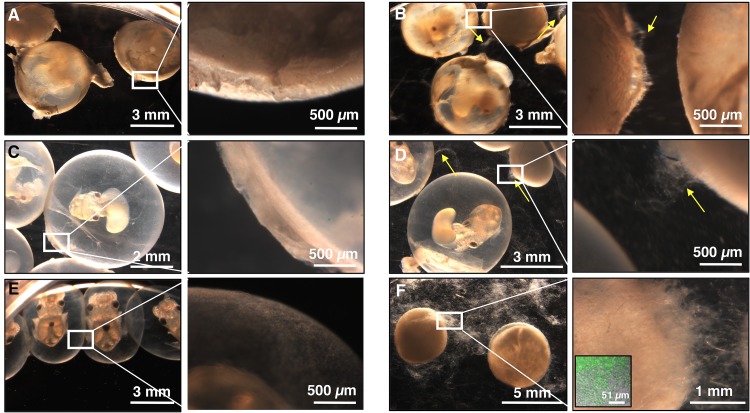
Embryos lacking jelly coats are more susceptible to fungal infection. Eggs either were left untreated (A, C, and E) or were challenged with F. keratoplasticum FSSC-2g at 10^4^ conidia/ml for 18 days (B, D, and F). Eggs left intact (A and B) or that were decapsulated, leaving only the jelly coat and developing embryos (C and D), were observed with few hyphae when challenged (B and D; yellow arrows), while eggs that were decapsulated and for which the jelly coat was removed, leaving only the embryo (E and F), were covered with fungal hyphae when challenged (F). Hyphae were stained with Syto9 nucleic acid stain and visualized with a confocal microscope (F, inset). (*n* = 4 trials, 10 eggs/treatment). Shown are representative images from a single trial.

To distinguish the functions of the bacterial community from potential embryonic factors that might have a protective effect, eggs were dissected into separate components and challenged with a conidial suspension of F. keratoplasticum FSSC-2g at 10^4^ conidia/ml (a concentration approximately an order of magnitude higher than probable environmental levels [8]) (Fig. 3B, D, and F). These eggs were not treated with antibiotics, leaving the egg bacterial community intact. By day 18 of embryogenesis, when the embryos were mature and near hatching, challenged whole eggs showed negligible signs of fungal infection (Fig. 3B), with only a few hyphae noted on the surface of the eggs and no apparent effect on the development of the embryos. Similar results were observed when the outer capsule was removed, leaving the JC and its bacterial community intact (Fig. 3D). However, when both the outer capsule and the JC were removed, leaving only the developing embryo within its yolk sac, eggs were heavily fouled by a fungal biomass and the embryos were not viable (Fig. 3F). Control eggs developed normally and were not fouled (Fig. 3A, C, and E). These experiments, along with the antibiotic treatments discussed above, suggest that the JC and its bacterial community provide protection against F. keratoplasticum.

### Symbiotic bacteria and their metabolites inhibit fungi.

To better understand the potential antifungal roles of specific bacterial strains that comprise the ANG and JC bacterial communities, 32 of 33 bacterial isolates were tested using a well diffusion inhibition assay against F. keratoplasticum FSSC-2g (one strain, *Ruegeria* ANG-S4, grew very slowly in culture and so was excluded from this assay but was included for extract assays [see below]). Three *Alphaproteobacteria* strains (*Leisingera* sp. strains ANG-S and ANG15 and *Labrenzia* sp. ANG18) and four *Gammaproteobacteria* strains (*Alteromonas* sp. JC21, *Vibrio* sp. JC34, and *Pseudoalteromonas* sp. strains JC22 and JC28) were strong inhibitors of F. keratoplasticum FSSC-2g, reducing the area of hyphal growth to 0% to 25% of that seen with the negative control (Fig. S5; see also Table S2a in the supplemental material). Ten strains showed moderate inhibition (26% to 50% growth), 11 strains showed weak inhibition (51% to 75% growth), and 4 strains exhibited minimal or no inhibition (≥76% growth) (Fig. S5; see also Table S2a). Together, our results show that 28 of 32 (87.5%) ANG/JC strains have the physiological potential to inhibit F. keratoplasticum.

10.1128/mBio.02376-19.5 FIG S5 ANG/JC bacterial isolates differentially inhibited F. keratoplasticum FSSC-2g. (A) Percent hyphal growth compared to the respective medium controls (seawater tryptone [SWT] or SWT with no glycerol [SWT_ng_]) was measured when conidia were plated in the presence of ANG/JC isolates. (B) A majority of ANG/JC isolates strongly (0% to 25% of control growth), moderately (26% to 50% of control), or weakly (51% to 75% of control) inhibited fungal growth. (C to H) Representative images of F. keratoplasticum hyphal growth on SWT medium (C), in the presence of cycloheximide (1,000 μg/ml) on SWT (D), with previous growth from isolate JC21 on SWT (E), JC21 on SWT_ng_ (F), ANG-S5 on SWT (G), and ANG-S5 on SWT_ng_ (H). Scale bars = 1 cm. Average results of three trials. Since bacterial secondary metabolite repression can occur in the presence of glycerol (68), we tested all of our strains under culture conditions with and without glycerol. Thirteen isolates gained activity (≥9% difference) when grown without glycerol, and 13 different isolates lost activity (≥9% difference; Table S2). Although the major carbon sources utilized by bacteria in the JC remain unclear, carbon catabolite repression in several antimicrobial-producing *Gammaproteobacteria*, *Firmicutes*, and *Actinobacteria* species has been reported previously (68). The effects of carbon source on secondary metabolite production are pathway specific, however, and the presence of glycerol has been shown to increase production of the antifungal nystatin in Streptomyces noursei (79). Because little is known about the chemistry of marine *Proteobacteria* and their secondary metabolite biosynthesis, various culture conditions should be tested in future studies to better explore the biosynthetic potential of ANG/JC bacteria. Download FIG S5, PDF file, 1.5 MB. Copyright © 2019 Kerwin et al. 2019 Kerwin et al. This content is distributed under the terms of the Creative Commons Attribution 4.0 International license.

10.1128/mBio.02376-19.9 TABLE S2 (A) Percentages of F. keratoplasticum FSSC-2g hyphal growth from well diffusion assay. (B) Percentages of F. keratoplasticum spp. (FSSC-2i, FSSC-2g, and FSSC-2d) and Candida albicans wild-type fungal growth from the 96-well liquid antifungal assay. Download Table S2, PDF file, 0.2 MB. Copyright © 2019 Kerwin et al. 2019 Kerwin et al. This content is distributed under the terms of the Creative Commons Attribution 4.0 International license.

**FIG 4:**
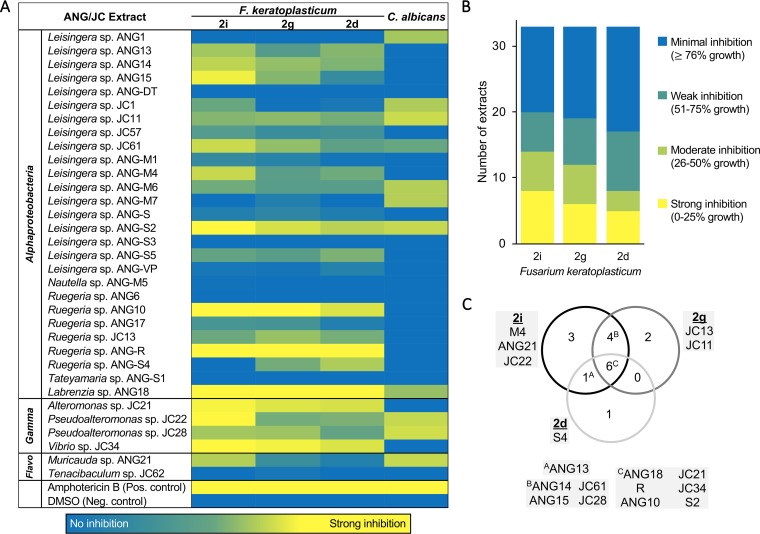
Bacterial secondary metabolites from the ANG and JC isolates demonstrate differential activity against fungal pathogens. (A) Many extracts from both ANG and JC bacterial isolates exhibited antifungal activity against one or more strains of Fusarium keratoplasticum and the human pathogen Candida albicans. Amphotericin B (4 μg/ml) was used as a positive control. Data shown are based on average percent growth values of technical triplicates from at least two experimental replicates. (B) Over 51% of ANG and JC bacterial extracts strongly or moderately inhibited the egg-isolated F. keratoplasticum strains, FSSC-2i and FSSC-2g, compared to the clinical F. keratoplasticum FSSC-2d isolate. (C) Venn diagram showing extracts that were active against each fusarial strain. Overall, six ANG and JC extracts exhibited antifungal activity against all three F. keratoplasticum strains, with other extracts showing activity against just one or two of the strains. Highlighted boxes indicate strain numbers listed in panel A.

To investigate the role of bacterial secondary metabolites in protecting the eggs from fungal fouling, 33 bacterial isolates from the ANG and JC were cultured, extracted, and tested in a 96-well liquid assay against three F. keratoplasticum strains: FSSC-2i (Hawaiian isolate), FSSC-2g (Connecticut laboratory isolate), and FSSC-2d (human clinical strain). In addition, extracts were tested against the yeast Candida albicans ATCC 18804, a common member of the human microbiota that can be a significant opportunistic pathogen. Overall, 8 of the 33 extracts exhibited strong inhibition (0% to 25% growth) against at least one F. keratoplasticum strain, an additional 9 extracts exhibited moderate inhibition (26% to 50% growth), and 5 extracts showed weak inhibition (51% to 75% growth) (Fig. 4; see also Table S2b). In total, 72.7% of the ANG and JC bacterial extracts showed antifungal activity, strongly suggesting a functional role of these symbionts in protecting embryos against fungal infection.

While six bacterial extracts (four from *Alphaproteobacteria* and two from *Gammaproteobacteria*) exhibited moderate to strong inhibition against all three strains of F. keratoplasticum, several extracts inhibited only one or two of the three strains (Fig. 4C). A greater number of bacterial extracts were classified as strongly or moderately active against FSSC-2i/2g than against FSSC-2d (Fig. 4B). Given that FSSC-2d is a clinical strain and not isolated from squid eggs, this differential activity may be due to adaptation of active metabolites to different fungal strains. Ten ANG and JC bacterial extracts (30.3%) also exhibited strong or moderate inhibition of C. albicans (Fig. 4A). The ANG and JC extracts often showed differential activity, with some active against only F. keratoplasticum (e.g., *Ruegeria* sp. strains ANG10 and ANG-R) and others active against only C. albicans (e.g., *Leisingera* sp. strains ANG1 and ANG-M7; Fig. 4A). Future research will focus on expanding these assays to testing other fungi to determine whether JC symbionts differentially inhibit a variety of fungi.

Overall, 7 of the 32 isolates (21.9%) and 8 of the 33 extracts (24.2%) exhibited strong inhibition against F. keratoplasticum. In other systems, such as the cnidarian hydra, interactions between multiple members of the epithelial bacterial community are necessary to protect the host from fusarial infections (3). Synergistic antifungal activity has not yet been determined in the bobtail squid system, but, given the complexity of the bacterial community found in both the ANG and JC, similar effects may be present.

Our experimental data demonstrate that ANG bacteria and JC bacteria and associated organic extracts from these strains inhibit fungi in culture. Bacterial secondary metabolite protection from fungi has been documented in several invertebrate systems. In the Oriental shrimp (Palaemon macrodactylus) and the American lobster (Homarus americanus), eggs are associated with protective bacteria that produce the antifungal compounds 2,3-indolinedione (isatin) and 4-hydroxyphenethyl alcohol (tyrosol), respectively (1, 2). When Hydra vulgaris was treated with antibiotics to remove the resident epithelial microbiota, the animals developed a detrimental infection of unidentified *Fusarium*, which was rendered ineffective by then complementing the treated hosts with members of their original bacterial community (3). Female beewolves apply “Candidatus *Streptomyces philanthi*” symbionts from antennal reservoirs to the brood chambers of their larvae, which are then incorporated into the larval cocoon to protect against environmental fungi. This protection is largely accomplished by the bacterial symbionts’ production of piericidin, streptochlorin, and nigericin derivatives (7, 32, 33). *Lagria* beetles are associated with vertically transferred *Burkholderia* strains, localized to accessory glands and deposited in eggs for defense (34). An antifungal polyketide, lagriamide, was isolated from over 28,000 beetle eggs, likely produced by an uncultured symbiont, Burkholderia gladioli (35). These examples demonstrate that diverse aquatic and terrestrial invertebrates utilize symbiotic bacteria to ward off fungal infections, and our work provides additional evidence to support the ideas of a similar functional role of ANG and JC bacteria in E. scolopes.

### Chemical networking analyses reveal potential antimicrobial metabolites.

To further understand the function of ANG- and JC-associated bacteria, we conducted experiments to identify antimicrobial secondary metabolites from ANG and JC bacterial isolates.

**FIG 5:**
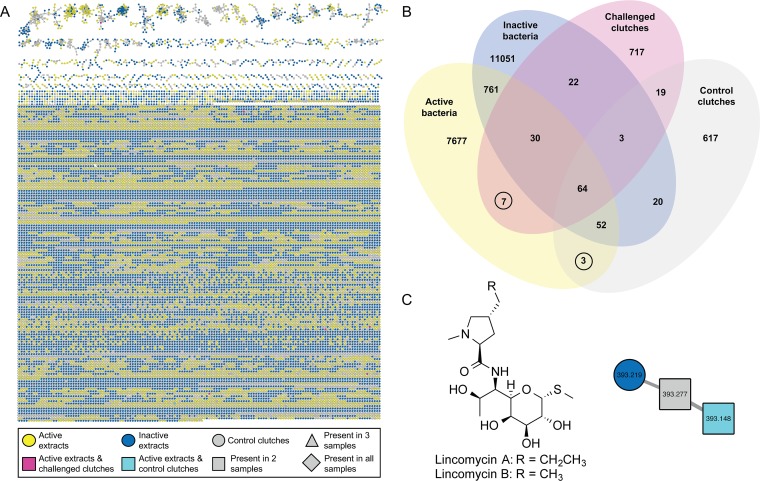
Comparative metabolomics identifies antimicrobial bacteria-derived secondary metabolites in egg clutches and active ANG and JC bacterial extracts. (A) LC-MS/MS molecular network of challenged and control clutches (see Fig. 6) with antifusarium active bacterial extracts (yellow circles) and inactive bacterial extracts (blue circles) resulted in a large, complex network (enlarged image of network provided in Fig. S7). Active bacterial extracts included only those with strong inhibition (0% to 25% fungal growth, *n* = 8), while inactive bacterial extracts included only those with minimal to no inhibition (≥76% fungal growth, *n* = 13). Parent masses are represented within each node, and the thickness of the edge is based on the cosine similarity score. (B) Ten MS features (circled) were prioritized for further investigation, including seven from challenged clutches that overlapped active bacterial extracts (pink squares) and three from control clutches that overlapped active bacterial extracts (teal squares). (C) Lincomycin B ([M + H]^+^ *m/z* 393.1480), a structural analogue of the FDA-approved antimicrobial drug lincomycin A, was identified in both the control clutches and the active extract, *Labrenzia* sp. ANG18 (identification confirmed via comparison with purchased standards). Lincomycin A was also found in the control clutch but was not detectable in the *Labrenzia* sp. ANG18 isolate.

**FIG 6:**
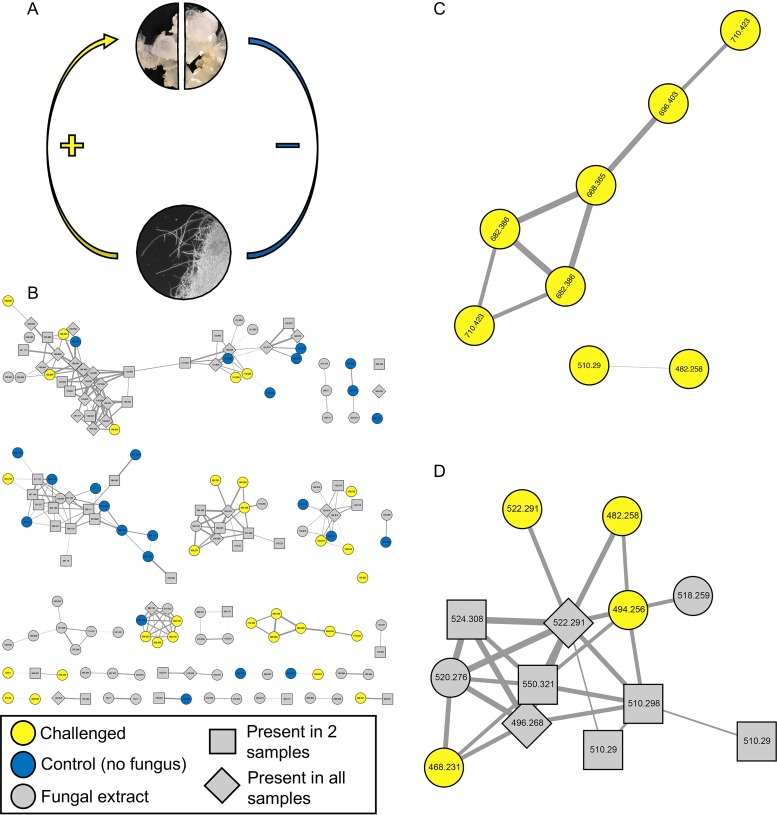
Comparative metabolomics identified specialized metabolite production by egg clutch-associated bacteria induced in the presence of Fusarium keratoplasticum. (A) The experimental design involved splitting egg clutches, challenging one portion with F. keratoplasticum FSSC-2g, and leaving one portion as an unchallenged control. No antibacterial treatment was added, leaving JC-associated bacteria potentially able to produce defensive metabolites. (B) LC-MS/MS molecular networking revealed numerous metabolites present only in challenged clutches (yellow circles), including two clusters with metabolites found only in challenged clutches (C). Metabolites were deprioritized if found only in control clutches (blue circles) and/or in F. keratoplasticum FSSC-2g extract (gray circles), with features found in two or more extracts shown in gray squares or diamonds. Parent masses are represented within each node, and the thickness of the edge is based upon the cosine similarity score. (D) Structurally distinct glycerophosphocholines (see the text) were also present in another cluster in this network, found in challenged clutches, control clutches, and/or F. keratoplasticum FSSC-2g. Several of the metabolites in this cluster were found only in the challenged clutches (yellow circles) or the F. keratoplasticum FSSC-2g extract (gray circles), while other metabolites in this cluster were found in both challenged and control clutches (gray squares) or in all three samples (gray diamonds) (see Table S3a).

Utilizing a combination of liquid chromatography-tandem mass spectrometry (LC-MS/MS) analyses and analyses of the biological activity of ANG and JC bacterial extracts (Fig. 4), we created an interaction-driven molecular network (Fig. 5A) (36) to compare compounds from bacterial extracts found to strongly inhibit at least one F. keratoplasticum strain (grouped as active) or those that failed to inhibit any of the F. keratoplasticum strains (grouped as inactive) with compounds also found in egg clutches, including both untreated clutches and clutches challenged with F. keratoplasticum (experimental design shown in Fig. 6A and discussed below). This network analysis enabled the identification of metabolites found in egg clutches that were also present in antifungal bacterial extracts (Fig. 5). As metabolites found in egg clutches are hypothesized to be derived from associated bacteria, mass spectral features that were found to cooccur in antifungal bacterial extracts and either the challenged or control clutches were prioritized (Fig. 5B), resulting in identification of 10 features of interest, 9 of which did not match any known metabolites. The remaining feature, *m/z* 393.1480, was present in the active extract of *Labrenzia* sp. ANG18 and the control clutches, and was found to be lincomycin B (Fig. 5C), a structural analogue of the FDA-approved antimicrobial drug lincomycin A. Upon subsequent examination of the raw LC-MS/MS data, lincomycin A was also found to be present in the control clutches but was not detected in the *Labrenzia* sp. ANG18 isolate. These results were confirmed via comparison with commercial standards (Fig. S6a).

10.1128/mBio.02376-19.6 FIG S6 (A) Lincomycin LC-MS/MS analyses. The data provided confirmation of the presence of lincomycin A and B (arrow) in the control clutch extract, including extracted ion chromatograms (EICs) for lincomycin B ([M + H]^+^ *m/z* 393.2059, *t*_r_ 2.63 min; panel A) and lincomycin A ([M + H]^+^ *m/z* 407.2240, *t*_r_ 2.95 min; panel B) (the lincomycin A standard splits into two peaks but the *t*_r_ and fragmentation patterns match for the standard peak and the matching peak in the control clutch; the peak at *t*_r_ 3.2 min was not consistent with lincomycin fragmentation). Lincomycin B was also detected in the bacterial isolate *Labrenzia* sp. ANG18, although lincomycin A was not detected in this isolate. Lincomycin A and B standards (Santa Cruz Biotechnology, Inc., Dallas, TX) were authenticated using ^1^H nuclear magnetic resonance (NMR) and HRMS prior to LC-MS/MS comparison. (B) Structures and sample sources of lincomycins, mycinamicins, and lyso-PAFs identified and/or tested here. Download FIG S6, PDF file, 0.4 MB. Copyright © 2019 Kerwin et al. 2019 Kerwin et al. This content is distributed under the terms of the Creative Commons Attribution 4.0 International license.

10.1128/mBio.02376-19.7 FIG S7 Enlarged image of LC-MS/MS molecular network of challenged and control clutches and active and inactive bacteria from the experiment described in the Fig. 5A legend. Download FIG S7, PDF file, 0.7 MB. Copyright © 2019 Kerwin et al. 2019 Kerwin et al. This content is distributed under the terms of the Creative Commons Attribution 4.0 International license.

10.1128/mBio.02376-19.10 TABLE S3 (A) Cluster of additional phosphocholines identified through the GNPS library from challenged clutches, control clutches, and F. keratoplasticum FSSC-2g extracts. (B) Mass spectral ion intensities*^a^* for nodes/compounds of interest from four separate challenged and control clutches. Download Table S3, PDF file, 0.1 MB. Copyright © 2019 Kerwin et al. 2019 Kerwin et al. This content is distributed under the terms of the Creative Commons Attribution 4.0 International license.

The lincomycins are the founding members of the lincosamide class of bacterial natural products first isolated in 1962 from the soil-derived actinomycete Streptomyces lincolnensis var. *lincolnensis* (37). Lincomycins have potent activity against Gram-positive bacteria (38), with lincomycin A (marketed as Lincocin) receiving FDA approval in 1967. Biosynthesis of the lincomycins involves unusual combinations of nonribosomal peptide synthetase (NRPS) and amino sugar enzymes to attach an *N*-methylated 4-propyl-l-proline to a methylmercapto eight-carbon sugar (39, 40). Commercially available lincomycins A and B were tested here for antifungal activity against F. keratoplasticum FSSC-2i and FSSC-2g and found to be inactive; however, several additional derivatives were preliminarily identified from this *Labrenzia* strain and future experiments will focus on isolating these derivatives and testing them for antifusarial activity. In addition, although these metabolites were inactive against F. keratoplasticum, community sequencing of the fungal biomass also detected the presence of bacteria (Fig. S4); thus, the presence of lincomycin A and lincomycin B in egg clutches may indicate a general antimicrobial response from the JC community. Regardless, the presence of this class of molecules in the egg clutches and in strain *Labrenzia* sp. ANG18 provides evidence for the functional potential of the ANG and associated bacteria in protecting squid eggs from infection.

In continued exploration of the metabolites involved in the antimicrobial protective effects of ANG- and JC-associated bacteria, we conducted an experiment to identify compounds induced in the presence of F. keratoplasticum (Fig. 6). Egg clutches were divided into two portions, with one part utilized as an untreated control and the other challenged with F. keratoplasticum FSSC-2g (Fig. 6A). These experiments did not involve antibiotic treatment, leaving JC-associated bacteria intact and potentially capable of producing defensive metabolites. Extracts from these two clutch portions as well as from a culture of F. keratoplasticum FSSC-2g (used to control for fungus-derived metabolites in the network) were networked (Fig. 6B). Several mass spectral features were found only in F. keratoplasticum-challenged clutches, including two of the highlighted clusters of metabolites (Fig. 6C). Further analyses of the larger highlighted cluster revealed the presence of molecules that share several structural characteristics with the known antimicrobials mycinamicins III, IV, and VI (Fig. S6b) (41–43). These shared structural characteristics include high-resolution MS data within acceptable limits and a distinctive in-source fragment consistent with a desosamine sugar moiety. Further testing of authenticated standards found that mycinamicins IV and VI exhibited weak antifungal activity against F. keratoplasticum FSSC-2g (73.4% and 68.6% growth, respectively), while mycinamicin III was inactive. Additional analyses of authenticated standards and egg samples by LC-MS/MS revealed differences in retention times that would suggest that these features were more similar to those of glycerophosphocholines, all known members of which had higher mass defects than the original assignments. Efforts are ongoing to unambiguously identify these features, including exploring possible effects of the gelatinous nature of the JC microenvironment on the physicochemical parameters (e.g., retention time). Regardless of their identification, production of the features associated with this cluster increased when eggs were challenged with F. keratoplasticum, suggesting an induced response of the JC community to fungal challenge.

Molecules from a second cluster induced in the presence of F. keratoplasticum (Fig. 6C), were putatively identified as glycerophosphocholines, similar to lyso-platelet activating factor (lyso-PAF) C:16 and lyso-PAF C:18. Commercial standards of these compounds were tested for activity against F. keratoplasticum FSSC-2i and FSSC-2g. Both compounds demonstrated antifungal activity with MIC values of 125 μg/ml. In subsequent LC-MS/MS analyses of additional clutches, we detected lyso-PAFs C:16 and C:18 in both challenged and control clutches. Further examination of the raw LC-MS/MS data confirmed that the original nodes were consistent with glycerophosphocholines but differed slightly from those of lyso-PAFs C:16 and C:18 and thus may represent related molecules from this large family of lipophilic compounds. In addition, other structurally distinct glycerophosphocholine derivatives were found in the network (Fig. 6D), although this cluster contains metabolites found in the challenged clutches, the control clutches, and/or the F. keratoplasticum extract (Table S3a). LC-MS/MS analyses of egg clutches from three additional experimental replicates confirmed the presence of the mycinamicin-like and glycerophosphocholine features in other clutches, with overall higher mean ion intensities in challenged clutches (Table S3b).

Previous reports of glycerophosphocholines, and specifically PAFs, indicated that these compounds are important defensive phospholipid signaling molecules that are deacetylated by acetylhydrolases to obtain lyso-PAFs (44, 45). These interconversions have been shown to be important for non-self-recognition, including antimicrobial defense in other marine host-microbe systems (46). Previous research suggests that bacteria capable of producing choline-containing molecules, such as glycerophosphocholines, are usually closely associated (e.g., symbiotic) with a eukaryotic host that supplies choline for the biosynthesis of these compounds (47, 48). The JC does contain membrane-like structures that separate each layer of the egg (17), although whether these structures contain cholines is not yet known. While our current studies do not reveal the origin of these glycerophosphocholines, a specific suite of these molecules appears to have altered production upon fungal challenge and may play an important role in egg defense.

The detection of antimicrobial compounds in both unchallenged eggs (lincomycins) and challenged eggs (mycinamicin-like compounds and/or glycerophosphocholines) suggests that eggs contain both constitutive and induced metabolites and thus have a broad potential to mount challenges to fouling and pathogens. Our interaction-driven network analyses revealed bioactive compounds in both control clutches and bacterial isolates, including such compounds as lincomycins, with known antibacterial activity. In addition, although identifications are still ongoing, the suite of glycerophosphocholines with altered production in the challenged clutches may represent a more generalized and inducible antimicrobial response of the symbiotic egg community to pathogens. Exploration of bacterial metabolites in the ANG will provide insight into whether compounds with antimicrobial activity may be deposited into the egg from the gland itself. Despite the presence of constitutive antimicrobials in the JC, our experimental data (Fig. 2 and 3) suggest that the presence of such compounds may not be enough to overcome fungal challenge and that metabolically active bacteria are likely also required for egg defense. Finally, the nine other unidentified MS features found in both clutches and active bacteria (Fig. 5) represent promising leads for the discovery of novel bioactive compounds.

### Conclusions.

Our research experimentally links a defensive function to the ANG/JC symbiotic bacterial community in the protection of squid eggs in the model cephalopod E. scolopes. Through experimental manipulation of the egg JC and its bacteria, we demonstrated that the JC is protective against microbial fouling. We further show that multiple bacterial members of the ANG/JC and their chemical extracts are capable of inhibiting the fungi Fusarium keratoplasticum and Candida albicans. Furthermore, we have identified several compounds, including lincomycin, mycinamicin-like compounds, and glycerophosphocholine derivatives as well as yet-to-be-identified compounds, that may play a role in egg defense. Although *Alphaproteobacteria* are underexplored for natural product production, interaction-driven networking revealed a number of potentially exciting bioactive leads. Because some host-microbe symbioses have evolved to select for bacterial symbionts that produce biologically active compounds (6), the ANG association may be a good source for antimicrobial drug discovery.

The ability of eggs from aquatic organisms to survive in an environment filled with potential biofoulers depends on the presence of one or more defensive mechanisms. The distribution of the ANG symbiosis in other cephalopods suggests that a similar function occurs in other members of this group. Questions regarding the function of the ANG symbiosis in cephalopods have been asked since the organ was first described in Doryteuthis pealeii in 1909 (20, 49, 50). In this study, we demonstrated that the JC bacterial community in E. scolopes prevents fungal fouling. Previous work demonstrated that members of the ANG bacterial community from E. scolopes and the squid D. pealeii can inhibit other bacteria (22, 51), and genomic analyses of ANG isolates suggested that these strains have a number of biosynthetic gene clusters that may be involved with the production of antimicrobials (52). Future work will focus on understanding whether there are conserved mechanisms of egg defense among cephalopods and whether specific pathogens are targeted. The few studies that have addressed ANG diversity suggested that *Alphaproteobacteria* and *Gammaproteobacteria* are present in the ANGs of other cephalopod species (13–15, 19). In the case of E. scolopes, the ANG is likely colonized by bacteria from the host’s environment (17). How ANG symbioses are established and maintained is another area of future research. For example, are members selected for based on their capacity to produce antimicrobial compounds and/or do other mechanisms, like competitive exclusion, play a role in shaping the consortium and/or egg defense? Selection of specific bacteria in binary host-microbe associations is common, but, even with complex consortia, certain bacterial taxa are often recruited (e.g., mammalian gut; 53–55). The mechanisms by which specific bacteria may colonize ANGs are currently unknown, but results from a study in the market squid D. opalescens suggest that the morphology of the nascent ANG is poised to recruit environmental bacteria (56). In the E. scolopes-V. fischeri light organ symbiosis, partners ensure specificity through a number of mechanisms (25), and it may be that similar colonization events occur in the ANG. Our previous work has also shown that specific taxa of the consortium are segregated within tubules of the ANG, suggesting that mechanisms are present to ensure colonization and growth of specific bacterial symbionts (16).

The phylogenetic and functional diversity of the members of the E. scolopes ANG symbiotic community offers a wide range of targets for future avenues of research, including the investigation of unexplored antimicrobial activity. Future research will examine whether certain bacteria and associated extracts work synergistically against potential biofoulers and pathogens. Cultivation efforts are also under way to isolate other bacteria in the ANG/JC community, such as *Verrucomicrobia* and *Flavobacteriia*, groups for which biosynthetic potential is unknown. In addition, a better understanding is needed of how the microenvironment of the JC (e.g., pH, jelly composition, and structural features such as the membrane-like structures; Fig. 1F) may facilitate delivery and efficacy of antimicrobial compounds. Overall, the bobtail squid-ANG association provides a tractable system to investigate functional specificity and drug discovery and to better understand the role of defensive symbioses in aquatic environments.

## MATERIALS AND METHODS

All experimental procedures involving animals were conducted in accordance with protocols approved by the Institutional Animal Care & Use Committee, Office of the Vice President for Research at the University of Connecticut (UConn), and in compliance with the Office of Animal Welfare, National Institutes of Health, and the Association for Assessment and Accreditation of Laboratory Animal Care International.

### Antibiotic clutch experiments.

Adult female Euprymna scolopes squid were collected from a protected sandflat in Maunalua Bay, Oahu, HI (21°26′3.36′′N, 157°47′20.78′′W) and were shipped to the University of Connecticut to be maintained in aquaria. Egg clutches laid in captivity by these squid were split into similarly sized groups and placed in aerated 0.22 μm filter-sterilized artificial seawater (FSASW). One group was treated with an antibiotic cocktail (25 μg/ml each of penicillin G, kanamycin, spectinomycin, streptomycin, and gentamicin, *n* = 5) and monitored over a 4-week period with daily water changes with fresh antibiotics added. A second group from the same clutch was left untreated and maintained under the same conditions without the addition of the antibiotic cocktail (*n* = 5). Clutches were maintained separately in a water bath at 25 to 27°C for the duration of experiments. The viability of each group was determined once hatching was complete by dividing the number of unhatched eggs by the number of total eggs (number of unhatched eggs added to the number of juveniles hatched). The health of a subset of hatched juveniles was checked by inoculating the water with the light organ symbiont Vibrio fischeri and by monitoring colonization using previously described methods (57). Percentages of hatch rates were compared between untreated and antibiotic-treated clutches using a paired two-tailed *t* test.

The effect of an antibiotic cocktail on embryo health was assessed by placing eggs under conditions that would not promote the growth of fungi. Clutches (*n* = 3 for each group) were placed in a laminar flow hood with FSASW and covered for the duration of the experiment. All equipment was UV sterilized for a minimum of 1 h prior to the addition of eggs. Clutches were aerated with pumps placed inside the laminar flow hood to prevent airborne contaminants from entering the water. FSASW was changed and fresh antibiotic cocktail was added every 2 to 3 days. Associated untreated controls from the same original clutches received no antibiotic cocktail. In a separate experiment, groups of clutches with or without antibiotic treatment were maintained at lower temperatures (15 to 20°C) to prevent fungal growth (*n* = 4). Neither growth nor germination of F. keratoplasticum FSSC-2g was observed under these conditions. The viability of each clutch was determined as described above.

To test antibiotic treatment under more natural conditions, clutches deposited by a wild-caught female bobtail squid were maintained in a tank with running unfiltered Hawaiian seawater and sand at Kewalo Marine Laboratory, Oahu, HI. These clutches (*n* = 3) were divided into two groups: treatment with 25 μg/ml of antibiotic cocktail and an untreated control. Clutches were maintained for 1 week in an aerated beaker with daily water changes and fresh antibiotics in the treatment group. After 1 week, both groups were transferred back to a tank with running seawater for the remainder of embryogenesis. The untreated group had 94% viability, while the antibiotic-treated section had 10% clutch viability and developed heavy fouling.

In order to test the effects of treatment using a different antibiotic, clutches were treated with chloramphenicol (20 to 25 μg/ml) or left untreated or treated with ethanol or water only (solvent controls for chloramphenicol). Laminar flow hood experiments were repeated as described above. Because chloramphenicol was found to have a negative impact on hatch rates (see Fig. S2 in the supplemental material), all subsequent experiments were conducted with the antibiotic cocktail.

### Fungal isolation and characterization.

Fusarium keratoplasticum FSSC-2g (CT 12-1807) was originally isolated from antibiotic treatment experiments as described above. F. keratoplasticum FSSC-2i (HI 66667) was isolated from an experiment conducted in natural Hawaiian seawater as described above. The outer capsule of an egg that did not hatch and a decapsulated egg containing a viable embryo from the antibiotic-treated section were both plated onto seawater tryptone (SWT [58]) for sampling of fungi.

Fungal cultures were further isolated on inhibitory mold agar with 0.05 g/liter gentamicin. Initial identification of fungal isolates occurred by analysis of morphologic characteristics on carnation leaf agar and DNA sequence analysis of the ITS-1 region of the rRNA gene. PCR was performed using two general fungal ITS primers (ITS-5 [5′-GGAAGTAAAAGTCGTAACAAGG-3′] and ITS-2 [5′-GGAAGTAAAAGTCGTAACAAGG-3′]) (29) and GoTaq polymerase (Promega, Madison, WI) under the following profile conditions: initial denaturation for 3 min at 95°C followed by 30 cycles of denaturation for 30 s at 95°C, annealing for 30 s at 62°C, and elongation for 1 min at 72°C. PCR products were sequenced in both directions and identified as F. keratoplasticum via BLASTn analysis at NCBI (https://www.ncbi.nlm.nih.gov/BLAST/) and RDP (http://rdp.cme.msu.edu/seqmatch).

Portions of three loci (*TEF1*, *RPB2*, and ITS plus large-subunit [LSU] ribosomal DNA [rDNA]) were used to identify three *Fusarium* strains used in the present study as F. keratoplasticum following a published protocol (29). Arabic numerals and lowercase roman letters, respectively, identify species and unique 3-locus haplotypes (Fig. S3). Maximum parsimony (MP) and maximum likelihood (ML) phylogenetic analyses were conducted with PAUP* 4.0b10 (59) and GARLI 2.01 (60), respectively. MP analyses were conducted using the heuristic search option, the tree bisection-reconnection (TBR) branch-swapping algorithm with MULPARS on, and 1,000 random sequence addition replicates. The CIPRES Science Gateway TeraGrid (https://www.phylo.org/) (61) was used to conduct the ML analysis employing the GTR + I + Γ model of molecular evolution. Clade support was assessed by 1,000 MP and ML bootstrap pseudoreplicates of the data.

### Egg component experiments.

A method developed for harvesting conidia of Fusarium oxysporum (62) was modified for use with F. keratoplasticum. Fusarium keratoplasticum FSSC-2g was grown on a rotary shaker for 3 to 5 days in SWT supplemented with 50 μg/ml chloramphenicol at 30°C. The culture was then strained through sterile gauze and centrifuged for 10 min at 3,200 × *g*. Conidia were rinsed twice with sterile water and repelleted after each wash followed by resuspension in one-fifth of the starting volume of sterile water. Conidia were quantified using a hemocytometer and correlated with the optical density at 530 nm (OD_530_) to determine a conversion factor of 3 × 10^6^ conidia/ml/OD_530_. Refrigeration for up to 1 week was found to have no effect on conidial viability.

E. scolopes eggs from one clutch were dissected using sterile forceps into three groups: intact eggs, eggs lacking outer capsule, and eggs lacking outer capsule and jelly coat (*n* = 4 separate trials; 8 to 10 eggs/group). Initial dissections were completed at day 5 of embryogenesis, as removal of the capsule and JC at earlier stages prevented embryo development. Each group was challenged with F. keratoplasticum FSSC-2g at 10^4^ conidia/ml over the course of embryogenesis, which is approximately an order of magnitude higher than eggs would be likely to encounter naturally (8). Eggs were maintained in FSASW with aeration, and water changes with fresh conidia were conducted every 2 to 3 days. For two of the four trials, two eggs from each group were removed and imaged every 3 to 4 days for the presence of fungal hyphae. For the other two trials, eggs were monitored visually for fungal hyphae over the course of the experiment (18 days).

### Transmission electron microscopy.

Fungally fouled eggs that had been treated with antibiotics were prepared for transmission electron microscopy (TEM) by first decapsulating the eggs and then fixing and embedding using established protocols (*n* = 3) (16, 17). Control eggs were not exposed to antibiotics (*n* = 3). Briefly, eggs were fixed (2.5% glutaraldehyde–2% paraformaldehyde solution) and stained with a solution of 1% osmium–0.8% potassium ferricyanide. Eggs were embedded in Spurr’s epoxy resin and sectioned on a Leica UCT Ultramicrotome (Leica Microsystems, Buffalo Grove, IL) into ultrathin sections (90 nm thickness). Samples were imaged on an FEI Technai Biotwin transmission electron microscope (FEI, Hillsboro, OR).

### Antibiotic-treatment effects on bacterial communities.

Antibiotic-treated clutches were maintained in a laminar flow hood to prevent infection as described above. FSASW was changed every 2 to 3 days, and fresh antibiotics were added. Five eggs from the same clutch (*n* = 5 clutches) were sampled on days 0, 10, and 15 of embryogenesis. JCs were isolated by surface sterilization in ethanol followed by homogenization in filter-sterilized squid Ringer’s solution (FSSR [16]) and were then plated on SWT to quantify culturable JC bacterial abundance. Differences in abundance between antibiotic-treated JCs and untreated JCs were analyzed via paired two-tailed *t* tests for each time point (Fig. 1L).

The fungal biomasses that formed on clutches treated with antibiotics were homogenized in FSSR (*n* = 12), and bacterial DNA from these samples was extracted using a DNeasy blood and tissue kit (Qiagen, Valencia, CA) according to the manufacturer’s protocol. DNA concentrations were determined using a Qubit double-stranded DNA (dsDNA) high-sensitivity assay (Thermo Fisher Scientific Inc., Waltham, MA) and averaged 1.13 ± 0.79 ng/μl. The V4 region of the 16S rRNA gene was sequenced on an Illumina MiSeq sequencer (Illumina, San Diego, CA, USA) following established protocols (17, 63, 64). Sequence data were analyzed using QIIME (65), and nonmetric multidimensional scaling (NMDS) plots of Bray-Curtis beta-diversity analyses were created in R using the VEGAN package (66) as previously described (17). Sequence data were compared to ANG/JC community data previously published under European Nucleotide Archive (ENA) project identifier (ID) PRJEB14655 with accession numbers ERS1498392 to ERS1498398, ERS1496666 to ERS1496676, and ERS1496678 to ERS1496722 (17).

### Well diffusion assay.

Bacterial isolates from the ANG and JC were obtained from previous studies (16, 22, 52) and this study (see Table S1 in the supplemental material) by homogenization in FSSR and plating serial dilutions on SWT and R2A media. Isolates from this study were identified to the genus level by Sanger sequencing of the 16S rRNA gene using universal primers (27F and 1492R [67]) and a BLASTn search of the 16S rRNA NCBI database.

10.1128/mBio.02376-19.8 TABLE S1 Bacterial and fungal strains used in this study. Download Table S1, PDF file, 0.1 MB. Copyright © 2019 Kerwin et al. 2019 Kerwin et al. This content is distributed under the terms of the Creative Commons Attribution 4.0 International license.

ANG/JC isolates were assayed for their ability to inhibit F. keratoplasticum FSSC-2g on SWT or SWT without glycerol (SWT_ng_) agar plates using an adaptation of the plate diffusion assay described by Fraune et al. (3). SWT_ng_ media were tested since glycerol can inhibit secondary metabolite production in some bacteria (68). Isolates were grown in 3.0 ml SWT or SWT_ng_ broth at 30°C with shaking at 120 rpm until cultures reached the stationary phase as measured by OD_600_. A sterile 6.0-mm-diameter borer was used to make wells in the agar, and 60 μl of each isolate culture was added to wells on separate plates. These plates were incubated for 48 h at 26°C to allow the isolate to form a biofilm around the walls of the well. F. keratoplasticum was then added to the wells at 10^4^ conidia/ml, and plates were further incubated under the same conditions for an additional 4 days. After incubation, plates were photographed and the area of visible hyphal growth was measured using FIJI image analysis software (69). Cyclohexamide (1,000 μg/ml) was used as a positive control, and SWT or SWT_ng_ broth (60 μl) was used as a negative control. Sterile water, which was used to dilute the F. keratoplasticum conidial stock in this assay, was also used as an additional negative control. For each bacterial strain, assays were performed using three separate experimental replicates with three technical replicates per experiment. Inhibition was reported as the percentage of hyphal growth compared to hyphal growth in the broth negative control. The total number of isolates which fell into each inhibition category (Fig. S5b) was determined from both SWT and SWT_ng_ assay results. If the degrees of inhibition differed between medium types, each isolate was counted only once and was included in the stronger-inhibition category.

### Culture and extraction of microbial isolates.

ANG and JC bacterial cultures were prepared for extraction using a modification of the previously described three-step culture protocol (22). All bacteria were cultured in SWT media. Small-scale cultures were prepared by inoculating 2 to 3 bacterial colonies into 5 ml of medium, medium-scale cultures utilized transfer of 1.5 ml of each small-scale culture into 50 ml of medium, and large-scale cultures involved transfer of 15 ml of each medium-scale culture into 500 ml of medium. All cultures were incubated for 3 days at 28°C with shaking at 200 rpm.

Large-scale bacterial cultures were extracted as previously described (22). Briefly, prewashed Diaion HP20 resin (Supelco, Bellefonte, PA, USA) (50 g, 10% [wt/vol]) was added to sonicated cultures and allowed to incubate for 24 h at room temperature with shaking (125 rpm). The resin and bacterial culture were then filtered, washed with water (discarded), and extracted with methanol (MeOH), dichloromethane (DCM), and acetone (American Chemical Society [ACS] grade; Sigma-Aldrich, St. Louis, MO, USA) (2 × 150 ml). Combined organic extracts were concentrated *in vacuo* and partitioned with ethyl acetate and water to remove residual aqueous material.

Fusarium keratoplasticum FSSC-2g was also cultured and extracted for use in metabolomics experiments. F. keratoplasticum FSSC-2g was grown on SWT agar as described below and transferred into 50 ml of fresh SWT medium. Prewashed Diaion HP20 resin (5 g, 10% [wt/vol]) was added and incubated and extracted as described above, using 2 × 30 ml of each solvent.

### 96-well liquid antifungal assays.

Bacterial extracts were tested for antifungal activity against F. keratoplasticum FSSC-2i, FSSC-2g, and FSSC-2d. Preparation of fungal inoculum followed previously described methodology (70), with each fungal isolate grown on SWT agar supplemented with 50 μg/ml of chloramphenicol at 28°C. Each culture was covered with 3 ml of medium, probed with a loop, and transferred into 10 ml of fresh medium. The fungal mixture was subjected to vortex mixing and allowed to settle, and the cell suspension below hyphae was transferred to a new Falcon tube. The cell suspension was adjusted to an OD_600_ of 0.15 to 0.17 and diluted 1:50 with medium to obtain a working solution.

F. keratoplasticum assays were performed in 96-well flat-bottom plates (Corning Costar; Corning, Kennebunk, ME, USA) according to Clinical and Laboratory Standards Institute (CLSI) reference method M38-A2 (71), with the following modifications. Each well consisted of 100 μl of diluted fungal inoculum, 98 μl of medium, and either 2 μl of a positive control (amphotericin B; final testing concentration, 4 μg/ml), a dimethyl sulfoxide (DMSO) negative control, or extract prepared in DMSO (final testing concentration, 500 μg/ml). Extracts and controls were tested in two separate experimental replicates with three technical replicates per experiment. Plates were read on a Synergy H1 hybrid reader (BioTek, Winooski, VT, USA) at 600 nm at 0 h, incubated at 28°C, and read again at 14 h. Percent growth was calculated in comparison with DMSO, the negative control.

Bacterial extracts were also tested against the human fungal pathogen Candida albicans ATCC 18804 in 96-well flat-bottom plates following CLSI method M27-A2 (72), with the following modifications. Yeast colonies were selected from an agar plate and inoculated into 5 ml of RPMI medium (Gibco) supplemented with 2% glucose and were then adjusted to an OD_600_ of 0.08 to 0.1 and diluted 1:10 with fresh medium to obtain a working solution. Each well consisted of 100 μl of diluted fungal inoculum, 98 μl of medium, and either 2 μl of a positive control (amphotericin B; final testing concentration, 2.5 μg/ml), a DMSO negative control, or extract prepared in DMSO (final testing concentration, 500 μg/ml). Extracts and controls were tested in two separate experimental replicates with three technical replicates per experiment. Plates were read on a Synergy H1 hybrid reader at 600 nm at 0 h, incubated at 35°C, and read again at 24 h.

### Fungal challenge of intact egg clutches.

Egg clutches were divided into two portions; one portion served as an untreated control, and the other was challenged by 10^4^ conidia/ml F. keratoplasticum FSSC-2g for 11 days in FSASW at room temperature with water changes and addition of fresh conidia every 2 to 3 days (*n* = 4; all clutches were analyzed via LC-MS/MS with one set of clutches used for the networking experiments described in the Fig. 6 legend). Experiments did not involve antibacterial treatment, leaving any JC-associated bacteria intact and potentially capable of producing defensive metabolites. Challenged and control eggs (5-ml volume each) were separately extracted with 2:1 DCM/MeOH (7 × 50 ml). Samples were sonicated, manually macerated with a metal spatula, and incubated for 10 min at room temperature, after which solvent was filtered, combined, and concentrated *in vacuo*.

### LC-MS/MS analyses and molecular networking.

Extracts from all clutches were analyzed via LC-MS/MS using a Waters Synapt G2-Si high-definition mass spectrometer (HDMS) coupled to a Waters Acquity ultra-high-performance (UPLC) liquid chromatography system. Separations utilized a Waters Acquity UPLC HSS T3 column (2.1 by 150 mm, 1.8-μm pore size, 100 Å) connected to an Acquity UPLC HSS T3 VanGuard precolumn (2.1 by 5 mm, 1.8-μm pore size, 100 Å). Samples were prepared at 1 mg/ml in a 1:1 mixture of methanol/water, and analytes were chromatographically separated using a linear 0.45 ml/min gradient under the following conditions: a 0.5-min hold at 95% solvent A (0.1% formic acid–water) and 5% solvent B (0.1% formic acid–acetonitrile), a 3.5-min ramp to 60% B, a 4-min ramp to 98% B, a 1-min hold at 98% B, a 0.2-min ramp back to 5% B, and a reequilibration hold at 5% B for 1.8 min.

Data were obtained using an 11-min Fast data-dependent acquisition (Fast DDA) method with a 0.1-s MS survey scan ranging from *m/z* 50 to 2,000, followed by five 0.1-s data-dependent MS/MS scans of ions with intensities greater than 1,000 as determined during the preceding scan event. Survey scans were acquired without collision energy (CE), and MS/MS scans were acquired using a linear two-stage low-mass (0-V to 20-V) and high-mass (10-V to 40-V) CE linear ramp. The instrument was operated in positive-resolution mode using the following parameters: 2 kV capillary voltage, 100°C source temperature, 20-V sampling cone, 800 liters/h desolvation gas flow, and 80-V source offset. LockSpray real-time mass correction was turned on to achieve optimal mass accuracy by monitoring 556.2771 *m/z* and 120.0813 *m/z* ions from simultaneous infusion of a 400 pg/μl leucine enkephalin solution (0.1% formic acid–50% water–50% methanol solution) with 0.1-s scans, a 10-s scan interval, and an average of 3 scans per correction.

Data were converted to mzML format using the ProteoWizard package (73) and processed in GNPS (Global Natural Products Social Molecular Networking [http://gnps.ucsd.edu [36]). For the challenged-clutch experiment, GNPS parameters were set as follows: minimum cosine similarity score of 0.65 with at least four matching peaks; parent mass tolerance of 1.0 Da; fragment ion tolerance of 0.02 Da; setting representing filter peaks in a 50-Da window turned off. The first network with ANG and JC bacterial extracts utilized the same parameters with the exception of having a fragment ion tolerance of 0.3 Da. Active extracts (0% to 25% growth of at least one *Fusarium* spp.) were grouped for these analyses and included *Labrenzia* sp. ANG18, *Leisingera* sp. ANG-S2, *Leisingera* sp. ANG15, *Ruegeria* sp. ANG-R, *Ruegeria* sp. ANG10, *Alteromonas* sp. JC21, *Pseudoalteromonas* sp. JC22, and *Vibrio* sp. JC34. Inactive extracts (≥76% growth of all three *Fusarium* spp.) were grouped separately from active extracts and included *Ruegeria* sp. ANG17, *Leisingera* sp. ANG-M1, *Leisingera* sp. ANG-S, *Leisingera* sp. ANG-VP, *Nautella* sp. ANG-M5, *Leisingera* sp. ANG-M7, *Leisingera* sp. ANG1, *Ruegeria* sp. ANG6, *Leisingera* sp. ANG-DT, *Leisingera* sp. ANG-S3, *Tateyamaria* sp. ANG-S1, and *Tenacibaculum* sp. JC62. Files were imported into either Cytoscape 2.8.3 (nodes arranged with FM3 layout) or Cytoscape 3.5.1 (nodes arranged using default layout) (74). High-resolution MS (HRMS) data corresponding to features of interest were used to search the METLIN database (75) for matching metabolites with a mass defect value within 20 ppm.

Standards of the mycinamicins (generously provided by David Sherman, University of Michigan), lincomycins (Santa Cruz Biotechnology, Inc., Dallas, TX), and lyso-PAFs (Cayman Chemical, Ann Arbor, MI) were used to determine presence in clutch extracts with a Waters Xevo G2-XS QToF mass spectrometer coupled to a Waters Acquity UPLC system. Separations utilized a Waters Acquity UPLC HSS T3 column (2.1 by 50 mm, 1.8-μM pore size, 100 Å) connected to an Acquity UPLC HSS T3 VanGuard precolumn (2.1 by 5 mm, 1.8-μM pore size, 100 Å). Conditions were identical to those described above, with the exception that MS/MS scans were acquired using linear two-stage low-mass (10-V to 4-V) and high-mass (40-V to 80-V) CE linear ramps. The instrument was operated in positive-sensitivity mode using the following parameters: 3-kV capillary voltage, 100°C source temperature, and 30-V sampling cone. LockSpray real-time mass correction used a simultaneous infusion of a 200 pg/μl leucine enkephalin solution.

### Data availability.

Bacterial sequences from the fungal biomasses were deposited in the ENA under project ID PRJEB23346.
