# Supplementary figures and images for: Shielding the Next Generation: Symbiotic Bacteria from a Reproductive Organ Protect Bobtail Squid Eggs from Fungal Fouling
Source: mBio. 2019 Oct 29;10(5):e02376-19. doi: 10.1128/mBio.02376-19 (PMC6819662; doi:10.1128/mBio.02376-19)

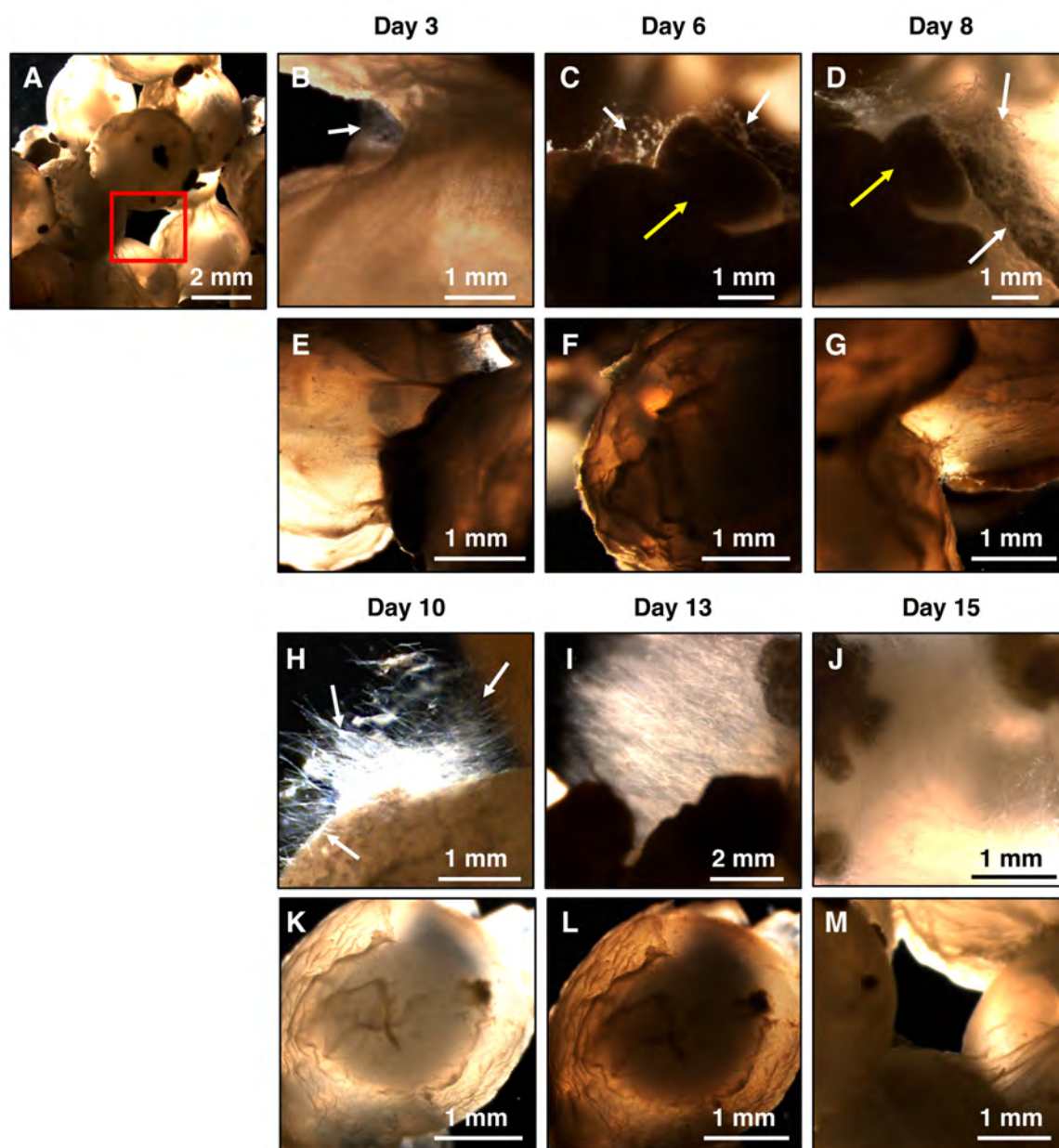

**Figure S1. A time series illustrating biofouling development over embryogenesis.**

Supplement: FIG S1 [file mBio.02376-19-sf001.pdf]

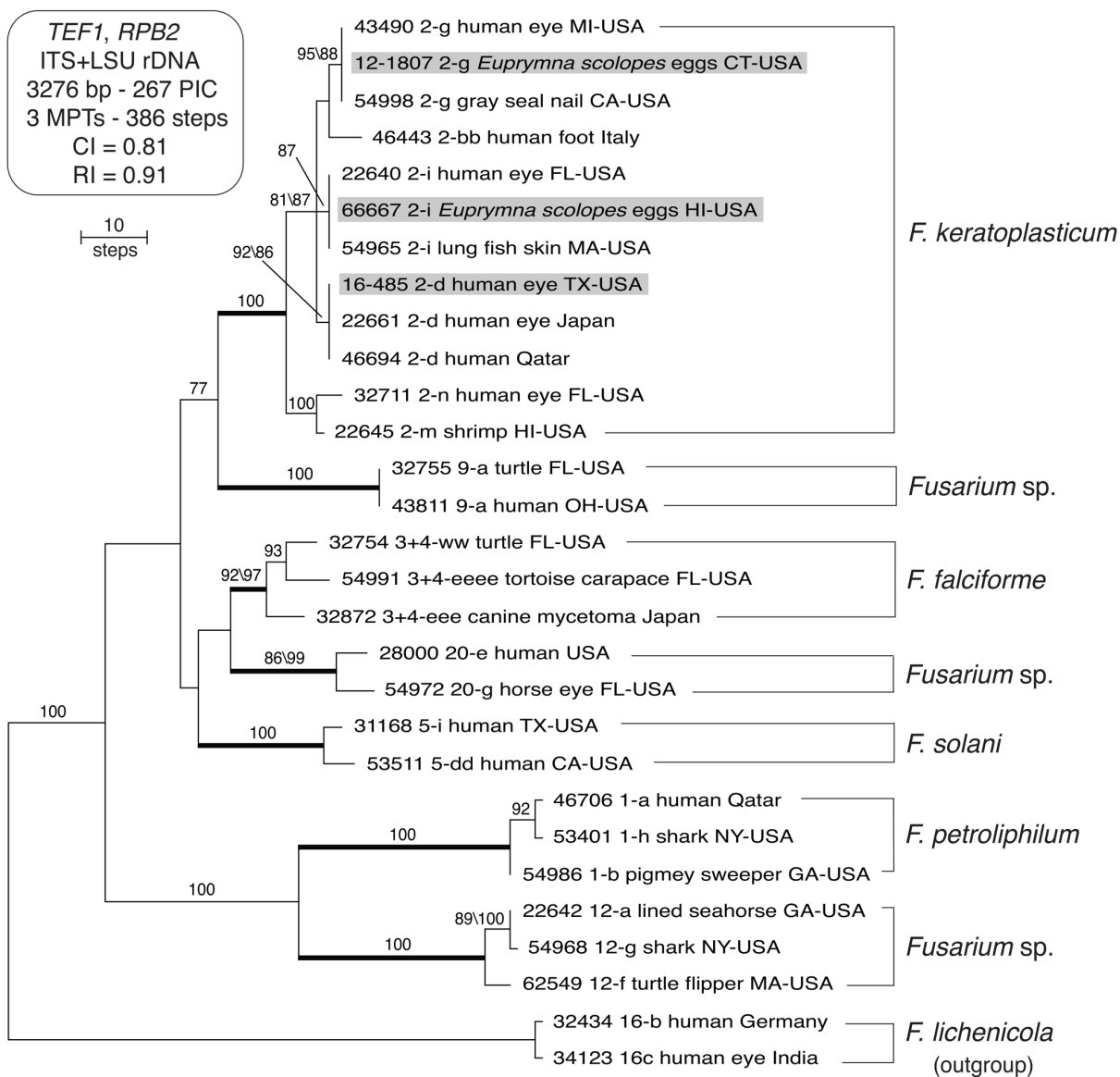

**Figure S3. Phylogeny of fungal isolates.**

Supplement: FIG S3 [file mBio.02376-19-sf003.pdf]

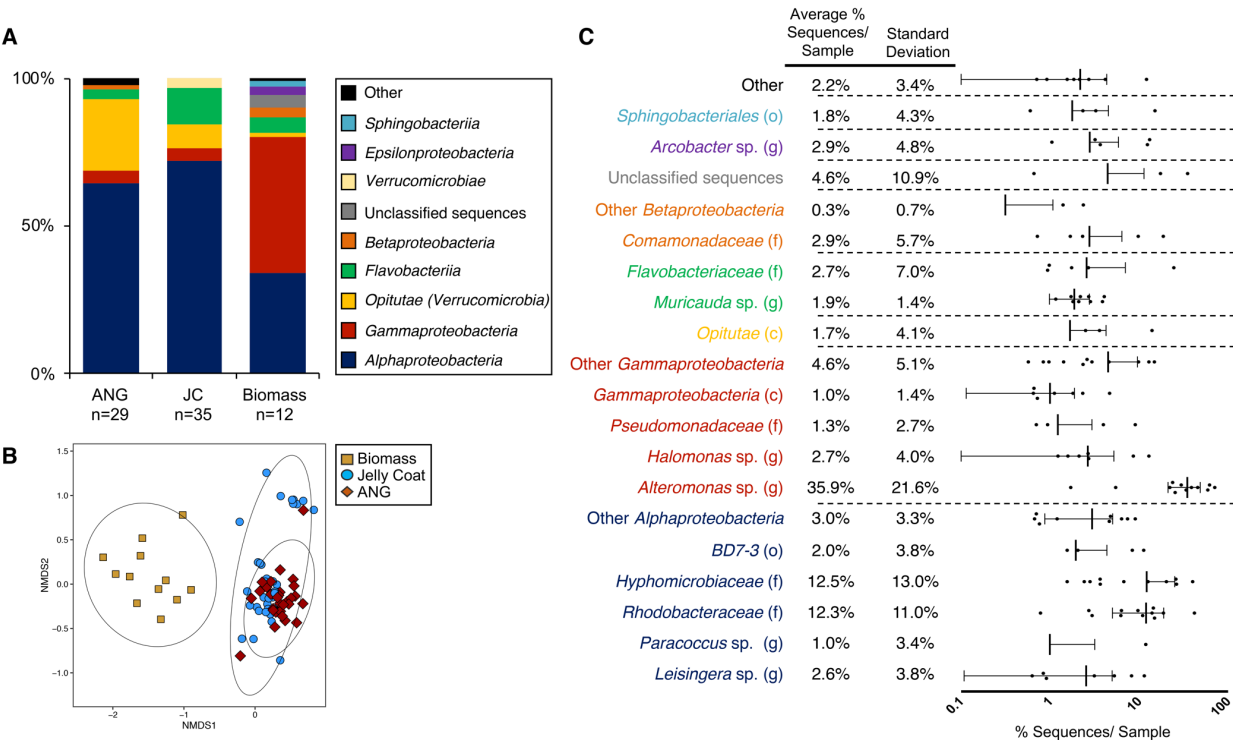

**Figure S4. Bacterial diversity in fungal biomass.**

Supplement: FIG S4 [file mBio.02376-19-sf004.pdf]

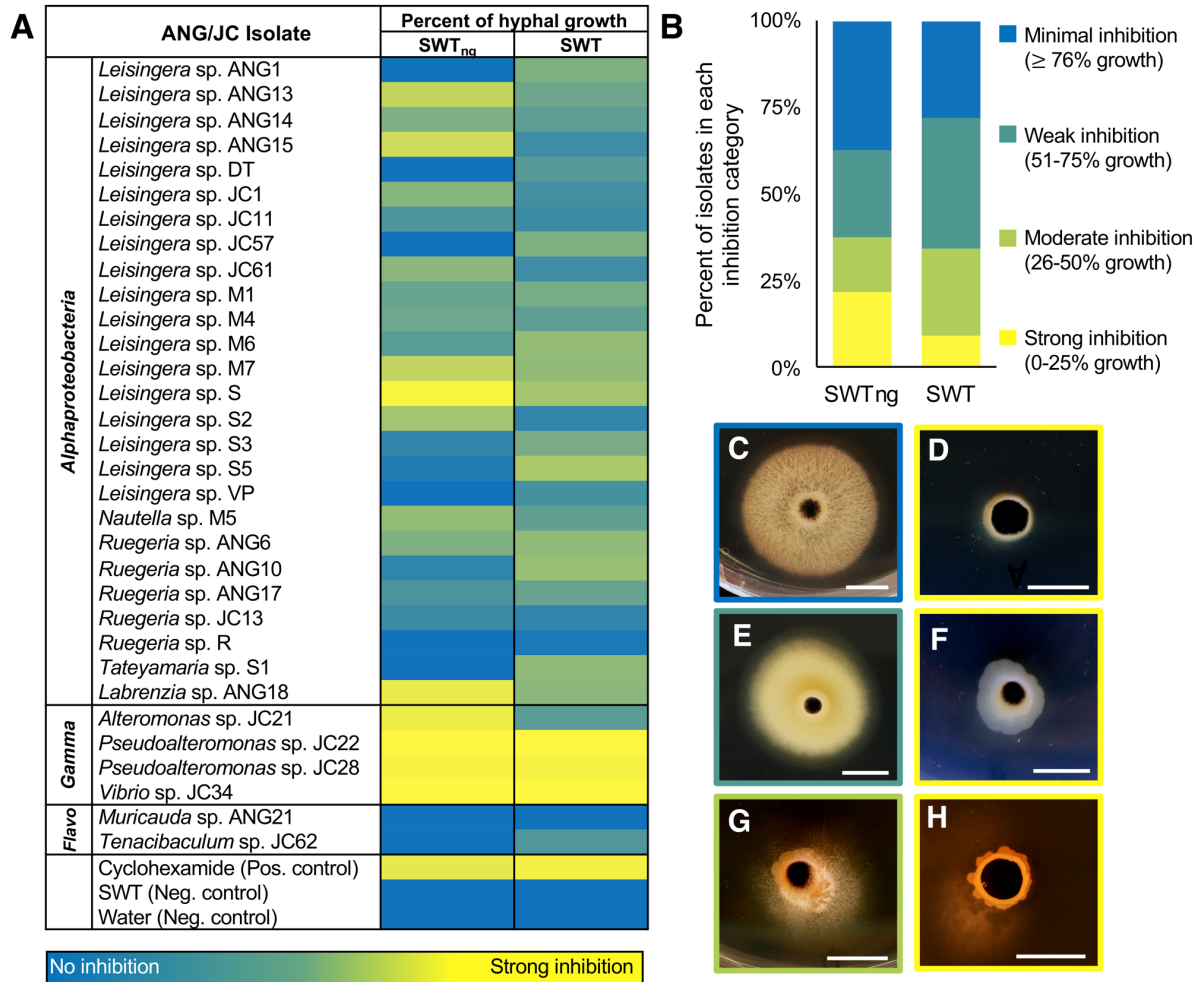

**Figure S5.** ANG/JC bacterial isolates differentially inhibited *F. keratoplasicum* FSSC-2g.

Supplement: FIG S5 [file mBio.02376-19-sf005.pdf]
