# Supplementary material for: Shielding the Next Generation: Symbiotic Bacteria from a Reproductive Organ Protect Bobtail Squid Eggs from Fungal Fouling
Source: mBio. 2019 Oct 29;10(5):e02376-19. doi: 10.1128/mBio.02376-19 (PMC6819662; doi:10.1128/mBio.02376-19)
Supplement: FIG S2 [file mBio.02376-19-sf002.pdf]

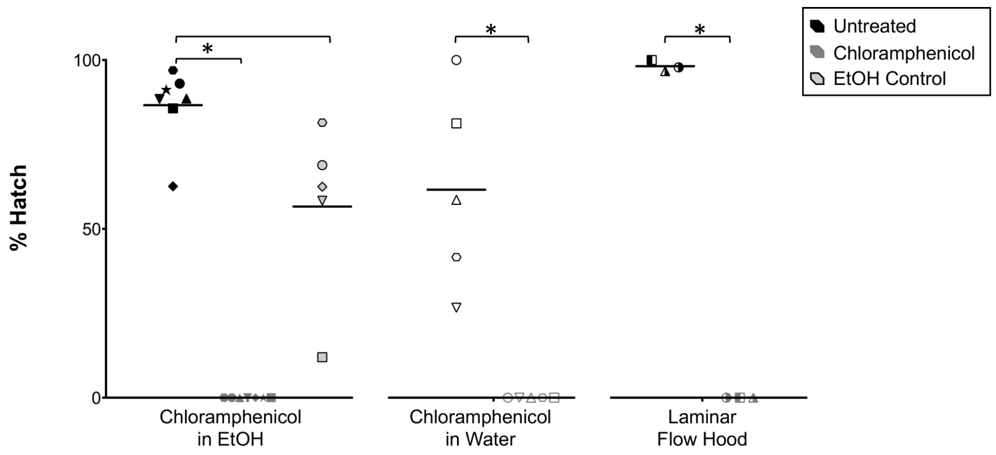

Experiment Environment	Treatment	# of clutches	Average Eggs/Clutch	Day of Biomass Appearance	Days Hatchlings Appeared	Average % Hatch
Laboratory	Chloramphenicol (20 μg/ml)	7	26	11-19	-	0%
	Ethanol Control	5	29	-	23-27	57% ± 26%
	Untreated	7	38	-	20-27	87% ± 11%
	Chloramphenicol (25 μg/ml)	5	37	10-12	-	0%
	Untreated	5	27	-	17-28	62% ± 30%
Laminar flow hood	Chloramphenicol (25 μg/ml)	3	45	-	-	0%
	Untreated	3	48	-	13 - 23	98% ± 2%

Figure S2. Chloramphenicol treatment of egg clutches resulted in fungal biomass.
