# Supplementary material for: Shielding the Next Generation: Symbiotic Bacteria from a Reproductive Organ Protect Bobtail Squid Eggs from Fungal Fouling
Source: mBio. 2019 Oct 29;10(5):e02376-19. doi: 10.1128/mBio.02376-19 (PMC6819662; doi:10.1128/mBio.02376-19)
Supplement: TABLE S2 [file mBio.02376-19-st002.pdf]

Table S2a. Percentages of *F. keratoplasticum* FSSC-2g hyphal growth from well diffusion assay.

	ANG/JC Isolate	Percent Hyphal Growth	
		SWT_{ng}	SWT
Alphaproteobacteria	*Leisingera* sp. ANG1	111.1±0.42	54±1.2
	Leisingera sp. ANG13	25.7±0.11	61.7±1.45
	Leisingera sp. ANG14	54.2±0.24	68±0.24
	Leisingera sp. ANG15	21±0.66	81±1.2
	Leisingera sp. ANG-DT	117.3±0.33	70.8±0.77
	Leisingera sp. JC1	51.4±0.45	79.2±1.1
	Leisingera sp. JC11	74.8±0.32	82.1±0.3
	Leisingera sp. JC57	116.4±2.33	53.9±0.06
	Leisingera sp. JC61	48.8±0.15	81±1.26
	Leisingera sp. ANG-M1	63.2±0.86	56.8±2.8
	Leisingera sp. ANG-M4	60.7±0.09	67.3±0.76
	Leisingera sp. ANG-M6	69.7±1.34	45.2±1.5
	Leisingera sp. ANG-M7	26.2±0.46	46±0.26
	Leisingera sp. ANG-S	2.3±0.23	38±1.1
	Leisingera sp. ANG-S2	39±0.28	86±0.62
	Leisingera sp. ANG-S3	86.7±0.13	56.1±0.15
	Leisingera sp. ANG-S5	92.9±0.27	35.3±0.7
	Leisingera sp. ANG-VP	125±0.96	77.7±1.2
	Nautella sp. ANG-M5	45.5±1.35	66.6±0.34
	Ruegeria sp. ANG6	54±0.24	46±1.7
	Ruegeria sp. ANG10	87.1±0.43	43±1.5
	Ruegeria sp. ANG17	75.4±0.2	64.3±0.96
	Ruegeria sp. JC13	82.6±0.46	86.7±0.81
	Ruegeria sp. ANG-R	105.9±1.5	95.6±0.26
	Tateyamaria sp. ANG-S1	112.1±1.5	47±1.2
	Labrenzia sp. ANG18	9.42±0.22	49.1±0.29
Gamma.	*Alteromonas* sp. JC21	7.6±0.23	69.7±0.44
	Pseudoalteromonas sp. JC22	0±0	0±0
	Pseudoalteromonas sp. JC28	3.1±0.1	4.4±0.11
	Vibrio sp. JC34	0±0	0±0
Flavo.	*Muricauda* sp. ANG21	155.1±0.63	110.8±0.42
	Tenacibaculum sp. JC62	132.7±0.31	73.9±1.4
Controls	Cyclohexamide (Pos. control)	11.2±0.1	5.1±0.21
	SWT (Neg. control)	100±0.68	100±0.46
	Water (Neg. control)	109.8±0.95	100.4±0.36

Percentages are an average of at least 3 trials ± the standard error of the mean. *Gamma.* (*Gammaproteobacteria*),*Flavo.*(*Flavobacteriia*).

Table S2b. Percentages of *F. keratoplasticum* spp. (FSSC-2i, FSSC-2g, and FSSC-2d) and *Candida albicans* wild type (Ca) fungal growth from the 96-well liquid antifungal assay.

	ANG/JC Isolate	Percent Fungal Growth			
		**FSSC-2i**	**FSSC-2g**	**FSSC-2d**	**Ca**
Alphaproteobacteria	*Leisingera* sp. ANG1	104.3±5.3	106.8±14.9	116.3±14.5	36.7±5.5
	Leisingera sp. ANG13	42.8±1.1	68.0±1.6	49.4±2.5	141.2±10.2
	Leisingera sp. ANG14	33.1±8.2	42.8±8.7	52.2±2.8	132.9±1.5
	Leisingera sp. ANG15	14.8±14.8	48.4±3.2	81.2±4.8	158.9±6.1
	Leisingera sp. ANG-DT	108.3±8.3	109.3±5.6	134.0±4.3	136.7±1.3
	Leisingera sp. JC1	64.0±9.5	99.6±7.5	94.6±11.1	33.3±9.8
	Leisingera sp. JC11	53.5±5.9	45.9±4.8	56.4±2.5	22.5±3.9
	Leisingera sp. JC57	71.4±6.8	79.5±11.4	76.0±5.3	116.3±8.9
	Leisingera sp. JC61	29.1±2.5	43.6±8.1	64.9±14.5	61.2±18.9
	Leisingera sp. ANG-M1	83.3±3.2	87.4±4.8	97.9±13.3	153.4±3.0
	Leisingera sp. ANG-M4	29.8±3.1	66.3±7.7	58.6±0.6	142.0±15.0
	Leisingera sp. ANG-M6	61.0±6.5	66.8±6.4	67.5±10.7	31.2±7.8
	Leisingera sp. ANG-M7	101.2±8.4	89.7±9.6	121.8±10.9	31.4±7.4
	Leisingera sp. ANG-S	90.7±7.1	84.3±5.6	91.5±6.2	152.0±5.1
	Leisingera sp. ANG-S2	-11.7±9.3	16.8±15.0	29.6±15.4	24.2±7.0
	Leisingera sp. ANG-S3	118.7±11.6	138.5±5.5	122.0±11.7	183.8±0.2
	Leisingera sp. ANG-S5	65.8±13.7	66.4±8.8	53.4±8.0	112.0±2.7
	Leisingera sp. ANG-VP	96.5±5.7	97.2±1.5	88.5±12.1	144.7±9.9
	Nautella sp. ANG-M5	98.8±12.3	106.5±4.1	115.5±1.2	165.1±10.7
	Ruegeria sp. ANG6	104.7±5.4	117.8±10.1	104.9±8.9	137.3±12.2
	Ruegeria sp. ANG10	8.6±13.7	-15.1±29.0	16.6±13.2	122.2±3.6
	Ruegeria sp. ANG17	75.5±0.5	74.6±6.5	89.9±0.1	102.7±1.8
	Ruegeria sp. JC13	62.9±5.6	41.1±12.1	55.3±10.4	146.3±10.2
	Ruegeria sp. ANG-R	-150.9±22.7	-230.0±3.7	-109.6±14.7	110.0±10.5
	Ruegeria sp. ANG-S4	118.2±2.6	55.9±3.4	34.1±1.4	168.7±3.3
	Tateyamaria sp. ANG-S1	134.3±8.3	125.1±8.8	134.7±14.7	163.9±0.2
	Labrenzia sp. ANG18	-23.3±13.2	-60.9±18.8	-18.9±13.9	42.4±14.3
Gamma.	*Alteromonas* sp. JC21	12.7±14.8	13.7±21.2	18.4±8.8	164.3±5.3
	Pseudoalteromonas sp. JC22	-89.6±3.2	55.5±8.0	53.3±14.6	24.7±7.4
	Pseudoalteromonas sp. JC28	42.3±0.5	39.3±8.1	62.6±9.5	19.8±4.2
	Vibrio sp. JC34	7.8±10.8	4.9±11.1	16.4±7.6	116.7±3.7
Flavo.	*Muricauda* sp. ANG21	36.5±11.7	78.8±5.9	89.0±8.4	24.3±3.0
	Tenacibaculum sp. JC62	116.3±0.7	96.0±6.4	123.9±1.4	127.0±6.0
Controls	Amp B (Pos. control)	9.7±4.2	0.3±0.3	2.5±1.4	2.0±0.4
	DMSO (Neg. control)	100±0.0	100±0.0	100±0.0	100±0.0

Percentages are an average of at least 2 experimental replicates performed in technical triplicate ± the standard error of the mean. *Gamma.* (*Gammaproteobacteria*), *Flavo.* (*Flavobacteriia*).
