# Supplementary material for: Shielding the Next Generation: Symbiotic Bacteria from a Reproductive Organ Protect Bobtail Squid Eggs from Fungal Fouling
Source: mBio. 2019 Oct 29;10(5):e02376-19. doi: 10.1128/mBio.02376-19 (PMC6819662; doi:10.1128/mBio.02376-19)
Supplement: FIG S6 [file mBio.02376-19-sf006.pdf]

S6a

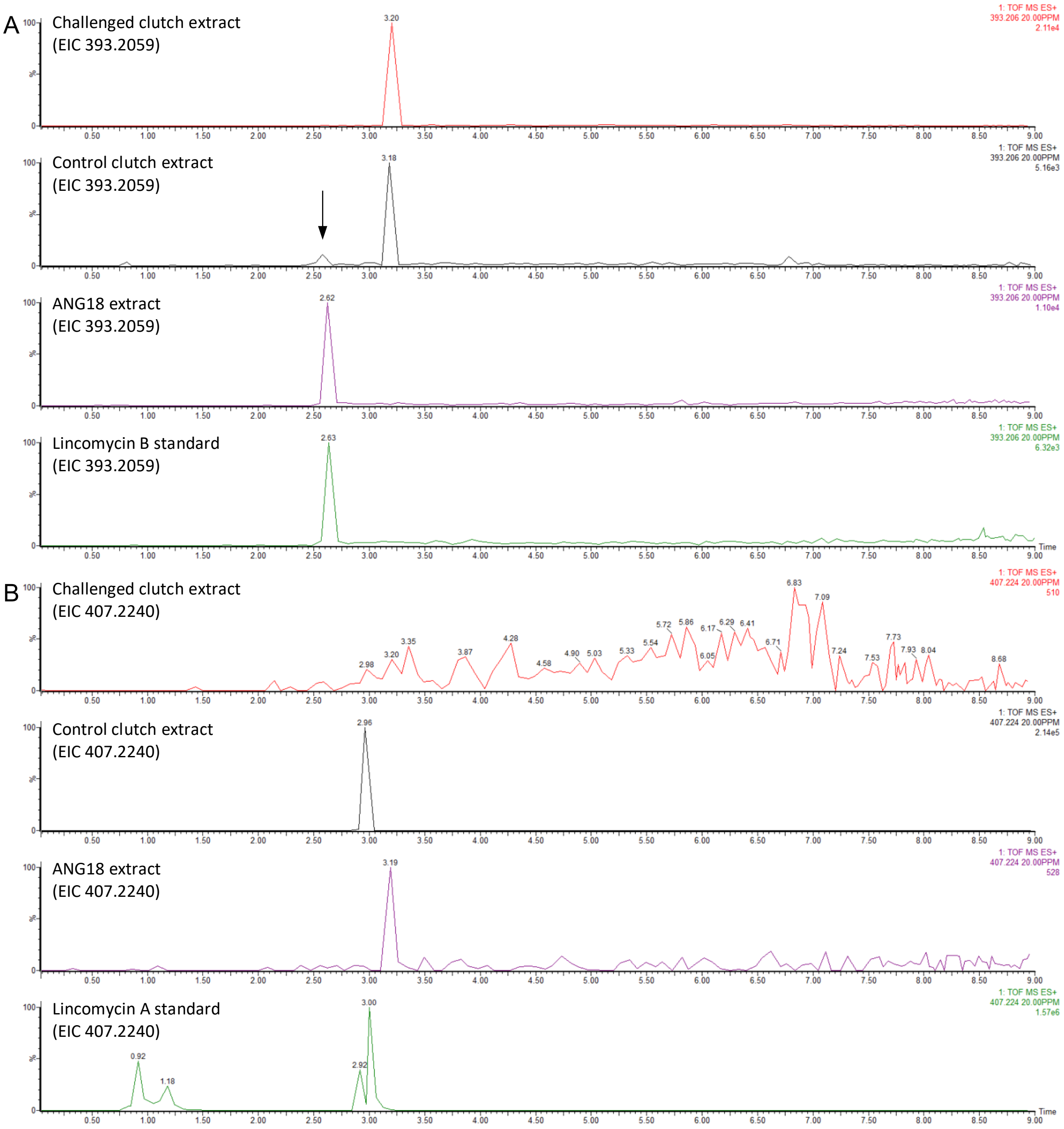
A
Challenged clutch extract
(EIC 393.2059)
1: TOF MS ES+
393.206 20.00PPM
2.11e4
3.20
Control clutch extract
(EIC 393.2059)
1: TOF MS ES+
393.206 20.00PPM
5.16e3
3.18
ANG18 extract
(EIC 393.2059)
1: TOF MS ES+
393.206 20.00PPM
1.10e4
2.62
Lincomycin B standard
(EIC 393.2059)
1: TOF MS ES+
393.206 20.00PPM
6.32e3
2.63
B
Challenged clutch extract
(EIC 407.2240)
1: TOF MS ES+
407.224 20.00PPM
510
Control clutch extract
(EIC 407.2240)
1: TOF MS ES+
407.224 20.00PPM
2.14e5
2.96
ANG18 extract
(EIC 407.2240)
1: TOF MS ES+
407.224 20.00PPM
528
3.19
Lincomycin A standard
(EIC 407.2240)
1: TOF MS ES+
407.224 20.00PPM
1.57e6
3.00
2.92
0.92
1.18
Time

S6b

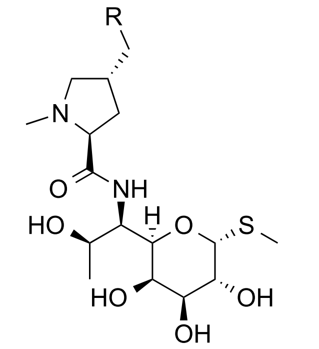

Lincomycin A: R = CH_2CH_3
Lincomycin B: R = CH_3

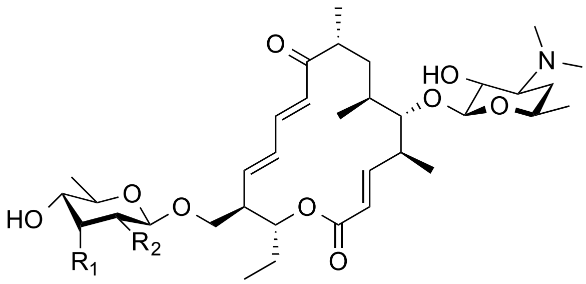

Mycinamicin III: R_1 = OH, R_2 = OMe
Mycinamicin IV: R_1 = OMe, R_2 = OMe
Mycinamicin VI: R_1 = OH, R_2 = OH

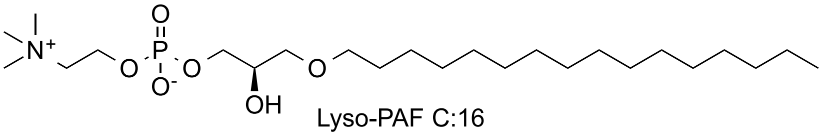

Lyso-PAF C:16

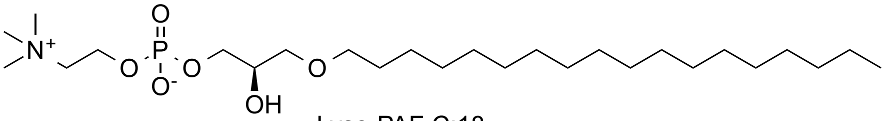

Lyso-PAF C:18

Compound/ Cluster	Sample(s)	Node mass[a]	Observed *m/z* $[M+H]^{+}$ [b]	Theoretical *m/z* $[M+H]^{+}$
Lincomycin A	Control clutch 1[c]	407.146	407.2267	407.2216
Lincomycin B	Control clutch 1[c] and *Leisingera* sp. ANG18	393.151	393.2054	393.2059
Fig. 6C, Cluster 1	Challenged clutch 1[c]	668.365	668.4087	
Fig. 6C, Cluster 1[d]	Challenged clutch 1[c]	682.386	682.4274	
Fig. 6C, Cluster 1	Challenged clutch 1[c]	696.403	696.4419	
Fig. 6C, Cluster 1[d]	Challenged clutch 1[c]	710.423	710.4592	
Fig. 6C, Cluster 2	Challenged clutch 1[c]	482.258	482.3294	
Fig. 6C, Cluster 2	Challenged clutch 1[c]	510.290	510.3581	
Mycinamicin III				682.4166
Mycinamicin IV				696.4323
Mycinamicin VI				668.4010
Lyso-PAF C:16				482.3610
Lyso-PAF C:18				510.3923

[a] consensus mass generated through GNPS
[b] experimental spectra obtained using Waters Synapt G2-Si, processed with MassLynx V4.1
[c] refers to control clutch 1 and challenged clutch 1 from Table S5
[d] two nodes with identical mass are found in this cluster, likely representing isomers

Figure S6. (a) Lincomycin LC-MS/MS analyses. (b) Structures and sample sources of lincomycins, mycinamicins, and lyso-PAFs identified and/or tested herein.
