# Supplementary material for: Shielding the Next Generation: Symbiotic Bacteria from a Reproductive Organ Protect Bobtail Squid Eggs from Fungal Fouling
Source: mBio. 2019 Oct 29;10(5):e02376-19. doi: 10.1128/mBio.02376-19 (PMC6819662; doi:10.1128/mBio.02376-19)
Supplement: FIG S7 [file mBio.02376-19-sf007.pdf]

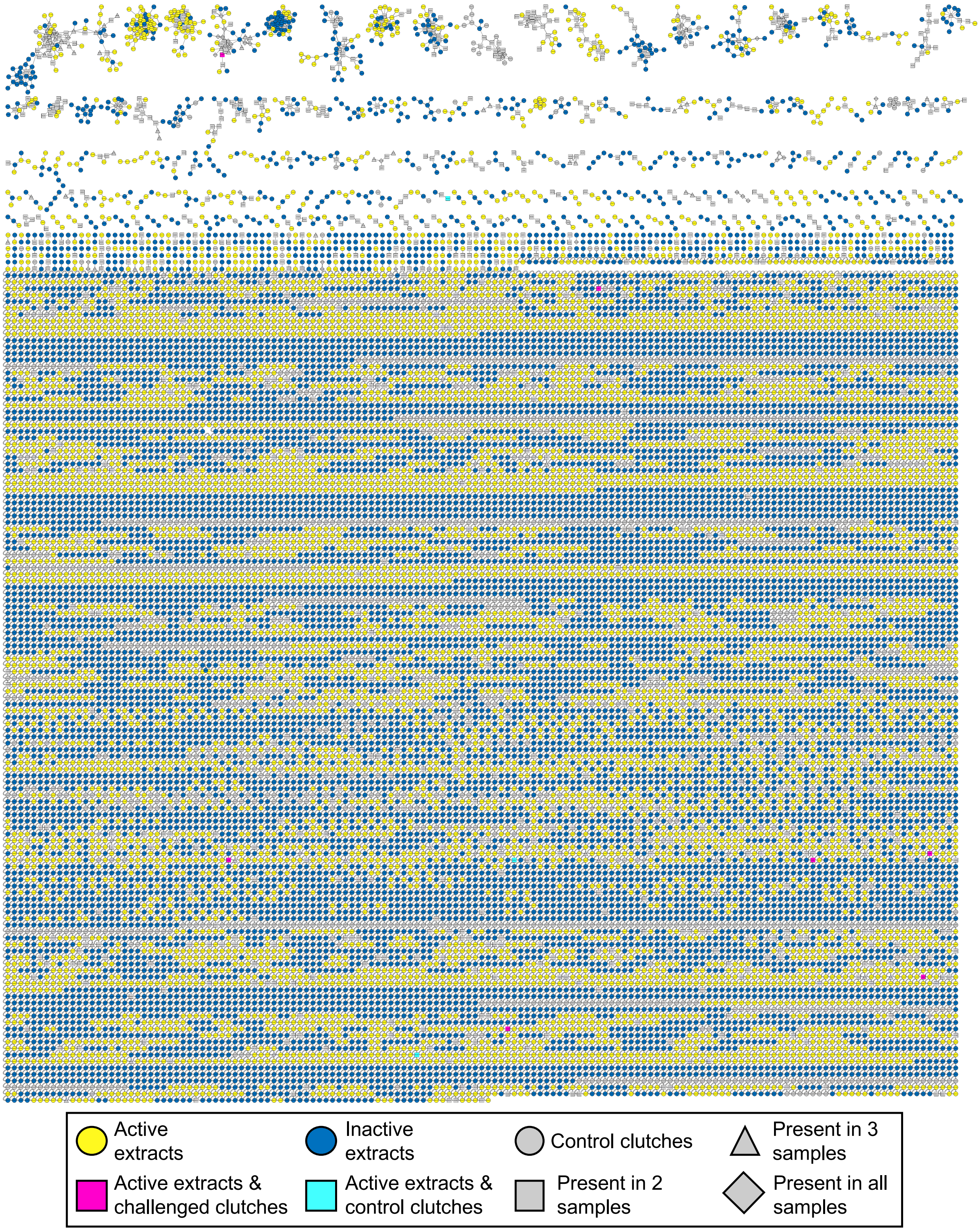

Figure S7. Enlarged image of LC-MS/MS molecular network of challenged and control clutches and active and inactive bacteria from Fig. 5A.
