# Supplementary material for: Shielding the Next Generation: Symbiotic Bacteria from a Reproductive Organ Protect Bobtail Squid Eggs from Fungal Fouling
Source: mBio. 2019 Oct 29;10(5):e02376-19. doi: 10.1128/mBio.02376-19 (PMC6819662; doi:10.1128/mBio.02376-19)
Supplement: TABLE S3 [file mBio.02376-19-st003.pdf]

Table S3a. Cluster of additional phosphocholines identified through the GNPS library from challenged clutches, control clutches, and *Fusarium keratoplasticum* FSSC-2g extracts.

Putative Identification[a]	Sample	Node mass[b]	Observed *m/z* $[M+H]^{+}$ [c]	Theoretical *m/z* $[M+H]^{+}$	Mass accuracy (ppm)
PC 0:0/14:0	Challenged clutches	468.231	468.3049	468.3085	7.6
PC 15:0/0:0	Challenged clutches	482.258	482.3198	482.3241	8.9
PC 16:1/0:0	Challenged clutches	494.256	494.3265	494.3241	4.8
PC 0:0/18:1[d]	Challenged clutches	522.291	522.3575	522.3554	4.0
PC 18:2/0:0	*F. keratoplasticum* FSSC-2g	520.276	520.3347	520.3398	9.7
PC 18:3/0:0	*F. keratoplasticum* FSSC-2g	518.259	518.3257	518.3252	0.9
PC 17:0/0:0[d]	Challenged and control clutches	510.290	510.3581	510.3554	5.3
PC 17:0/0:0[d]	Challenged and control clutches	510.290	510.3581	510.3554	5.3
PC 20:1/0:0	Challenged and control clutches	550.321	550.3832	550.3867	6.4
PC 17:0/0:0[d]	Challenged and control clutches	510.298	510.3581	510.3554	5.3
PC 0:0/18:0	Challenged and control clutches	524.308	524.3744	524.3711	6.4
PC 0:0/16:0	All 3	496.268	496.3372	496.3398	5.2
PC 0:0/18:1[d]	All 3	522.291	522.3575	522.3554	4.0

[a] features could represent one of several related isomers
[b] consensus mass generated through GNPS
[c] experimental spectra obtained using Waters Synapt G2-Si, processed with MassLynx V4.1
[d] several nodes with similar masses were found in this cluster, likely representing isomers

Table S3b. Mass spectral ion intensities[a] for nodes/compounds of interest from four separate challenged and control clutches.

	Node/Compound							
	Lyso-PAF-like				Mycinamicin-like			
**Sample**	**482.258**	**510.290**	**C:16[b]**	**C:18[b]**	**668.365**	**682.386**	**696.403**	**710.423**
Control clutch 1[c]	2.62e3[d]	4.60e3	9.25e4	1.77e4	3.59e3[d]	2.39e3[d]	1.61e3[d]	2.08e3[d]
Challenged clutch 1[c]	3.45e3	7.58e3	9.23e4	6.60e4	1.24e4	6.43e3	1.20e4	1.03e4
Fold change (challenged)	1.3	1.6	1.0	3.7	3.5	2.7	7.5	5.0
Control clutch 2	3.50e4	4.47e4	6.27e5	3.45e5	9.76e3	6.12e3	1.33e3	1.65e3
Challenged clutch 2	2.25e4	5.29e4	3.55e5	3.46e5	2.76e4	8.75e4	6.58e3	7.17e3
Fold change (challenged)	0.6	1.2	0.6	1.0	2.8	14.3	4.9	4.3
Control clutch 3	4.50e4	2.55e4	5.33e5	3.55e5	4.24e4	3.44e3	9.05e3	7.21e3
Challenged clutch 3	8.50e4	3.60e4	2.76e5	3.39e5	1.52e4	1.48e4	2.19e3	4.06e3
Fold change (challenged)	1.9	1.4	0.5	1.0	0.4	4.3	0.2	0.6
Control clutch 4	3.13e4	1.73e4	2.79e5	5.50e4	3.06e4	1.28e4	4.79e3	3.89e3
Challenged clutch 4	5.51e4	7.49e3	1.95e5	2.75e5	1.41e4	7.46e3	4.41e3	2.16e3
Fold change (challenged)	1.8	0.4	0.7	5.0	0.5	0.6	0.9	0.6
Mean fold change (challenged; ± SEM)	1.4±0.3	1.2±0.3	0.7±0.1	2.7±1.0	1.8±0.8	5.5±3.0	3.4±1.7	2.6±1.2

[a] Ion intensities were obtained from raw MS spectra at peak apex and represent semi-quantitative analysis

[b] Lyso-PAF C:16 and lyso-PAF C:18

[c] Clutch 1 was used for GNPS molecular networking in Fig. 6

[d] Below limit for MS/MS and thus not detected in GNPS although found to be present via MassLynx during MS scan
