# Supplementary material for: Shielding the Next Generation: Symbiotic Bacteria from a Reproductive Organ Protect Bobtail Squid Eggs from Fungal Fouling
Source: mBio. 2019 Oct 29;10(5):e02376-19. doi: 10.1128/mBio.02376-19 (PMC6819662; doi:10.1128/mBio.02376-19)
Supplement: TABLE S1 [file mBio.02376-19-st001.pdf]

Table S1. Bacterial and fungal strains used in this study.

Strain	Characteristics	Source
Fusarium keratoplasticum FSSC-2g	CT isolated strain	This study
Fusarium keratoplasticum FSSC-2i	HI isolated strain	This study
Fusarium keratoplasticum FSSC-2d	Texas isolated strain	Clinical isolate; this study
Candida albicans ATCC 18804	Type strain	American Type Culture Collection (ATCC)
Leisingera sp. ANG1	Isolate from *E. scolopes* ANG	Collins and Nyholm 2011, Collins et al., 2015
Leisingera sp. ANG13	Isolate from *E. scolopes* ANG	This study
Leisingera sp. ANG14	Isolate from *E. scolopes* ANG	This study
Leisingera sp. ANG15	Isolate from *E. scolopes* ANG	This study
Leisingera sp. ANG-DT	Isolate from *E. scolopes* ANG	Collins et al. 2015
Leisingera sp. ANG-M1	Isolate from *E. scolopes* ANG	Collins et al. 2015
Leisingera sp. ANG-M4	Isolate from *E. scolopes* ANG	Collins et al. 2012
Leisingera sp. ANG-M6	Isolate from *E. scolopes* ANG	Collins et al. 2015
Leisingera sp. ANG-M7	Isolate from *E. scolopes* ANG	Collins et al. 2015
Leisingera sp. ANG-S	Isolate from *E. scolopes* ANG	Collins et al. 2015
Leisingera sp. ANG-S2	Isolate from *E. scolopes* ANG	Collins et al. 2012
Leisingera sp. ANG-S3	Isolate from *E. scolopes* ANG	Collins et al. 2015
Ruegeria sp. ANG-S4	Isolate from *E. scolopes* ANG	Collins et al. 2015
Leisingera sp. ANG-S5	Isolate from *E. scolopes* ANG	Collins et al. 2015
Leisingera sp. ANG-VP	Isolate from *E. scolopes* ANG	Collins et al. 2015
Leisingera sp. JC1	Isolate from *E. scolopes* JC	Gromek et al. 2016
Leisingera sp. JC11	Isolate from *E. scolopes* JC	This study
Leisingera sp. JC57	Isolate from *E. scolopes* JC	This study
Leisingera sp. JC61	Isolate from *E. scolopes* JC	This study
Labrenzia sp. ANG18	Isolate from *E. scolopes* ANG	This study
Nautella sp. ANG-M5	Isolate from *E. scolopes* ANG	Collins et al. 2012
Ruegeria sp. ANG6	Isolate from *E. scolopes* ANG	This study
Ruegeria sp. ANG10	Isolate from *E. scolopes* ANG	This study
Ruegeria sp. ANG17	Isolate from *E. scolopes* ANG	This study
Ruegeria sp. ANG-R	Isolate from *E. scolopes* ANG	Collins et al. 2015
Ruegeria sp. JC13	Isolate from *E. scolopes* ANG	This study
Tateyamaria sp. ANG-S1	Isolate from *E. scolopes* ANG	Collins et al. 2015
Muricauda sp. ANG21	Isolate from *E. scolopes* ANG	Gromek et al. 2016
Tenacibaculum sp. JC62	Isolate from *E. scolopes* JC	This study
Altermonas sp. JC21	Isolate from *E. scolopes* JC	This study
Pseudoaltermonas sp. JC22	Isolate from *E. scolopes* JC	This study
Pseudoaltermonas sp. JC28	Isolate from *E. scolopes* JC	This study
Vibrio sp. JC34	Isolate from *E. scolopes* JC	This study
